# Exploring the gap between theory and experiment at the three-phase contact line of polystyrene droplets on soft PDMS

**DOI:** 10.1038/s41598-025-30195-y

**Published:** 2025-12-09

**Authors:** Khalil Remini, Leonie Schmeller, Dirk Peschka, Barbara Wagner, Ralf Seemann

**Affiliations:** 1 Department of Experimental Physics, Saarland University, 66123 Saarbrücken, Germany; 2 Weierstrass Institute for Applied Analysis and Stochastics, 10117 Berlin, Germany

**Keywords:** Materials science, Physics

## Abstract

The shape of liquid polystyrene (PS) droplets obtained via the dewetting of nanometer thin PS films from soft viscoelastic polydimethylsiloxane (PDMS) substrates are investigated. For a range of droplet sizes and substrate elasticities we measure the profiles of all the interfaces by combining lift-off techniques with atomic force microscopy and compare them to the predictions of fully time-dependent sharp-interface models for the PS/PDMS system, that are derived through energy minimization methods and allow to follow the dewetting dynamics towards their equilibrium states. Our analysis shows that there is a thin layer of uncrosslinked PDMS molecules that cloaks the PS droplets. By incorporating the effect of cloaking into the surface energies of our theoretical model, the experimental droplet and substrate profiles are shown to be in excellent quantitative agreement for all considered droplet sizes and substrate elasticities. Interestingly, our comparisons also establish small but systematic discrepancies between the experimental results and the theoretical predictions in the vicinity of the three-phase contact line. These discrepancies tend to increase for softer substrates and smaller droplets. Our analysis shows that global variations in system parameters, such as surface tension and elastic shear modulus, cannot account for these differences but instead point to a locally larger elastocapillary length, whose possible origins we investigate in detail.

## Introduction

Compared to the rather mature understanding of wetting and dewetting phenomena on rigid solid substrates, the physics of wetting on soft adaptive substrates lags behind due to a number of coupled physical phenomena that are involved. This includes the conceptual difference between surface tension and the free surface energy for solid surfaces that was pointed out in the pioneering work of Shuttleworth^1^, the dissipation in a microscopic *wetting ridge* that can influence the macroscopic dynamics by *viscoelastic braking*, which was introduced by Carré et al.^2^, and the time-dependent poroelastic relaxation of the soft solids^3^. It is only in recent years that an increasing number of theoretical and experimental studies have addressed the complex nature of these adaptive processes, in particular in the vicinity of a moving three-phase contact line (TPCL), e.g.^4–7^.

The most important quantity that governs *classical elastocapillarity*, i.e., static force balances between elasticity and capillarity at wetting interfaces and especially at the TPCL, is the elastocapillary length

1 $$\begin{aligned} \lambda _\textrm{c}= \frac{\gamma }{G}, \end{aligned}$$

measuring the length scale below which capillary effects usually dominate over elastic ones, where $\gamma$ is the surface tension of the wetting fluid and *G* the elastic shear modulus of the soft solid. For soft wetting on viscoelastic substrates, different regimes exist depending on the relative magnitude of the elastocapillary length compared with molecular scales $a \sim 10^{-9}\,\text {m}$, radius of the drop base *R*, and substrate thickness *H*.

**Fig. 1:**
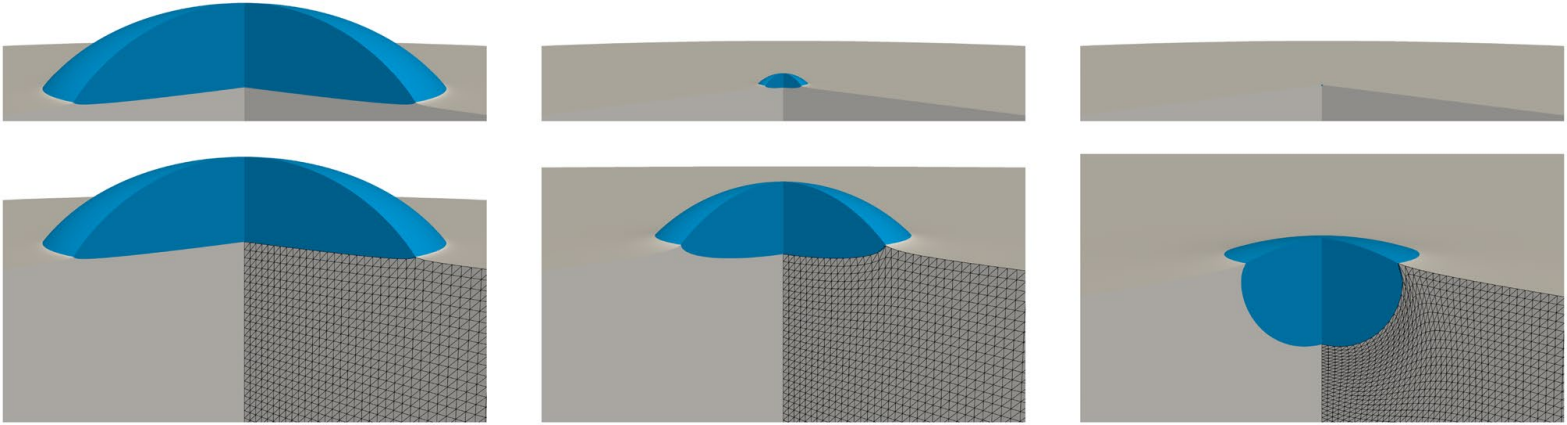
Axisymmetric stationary liquid droplets (blue) on an elastic substrate (gray) with height $H = 7~\upmu \text {m}$, computed using finite element simulations. The droplet radii are (left) $R \approx 2.5~\upmu \text {m}$, (middle) $R \approx 300~\text {nm}$, and (right) $R \approx 13~\text {nm}$, based on SG186 material parameters with a capillary length $\lambda _\textrm{c}= 80~\text {nm}$. The upper panel shows all droplets at a fixed scale of $3~\upmu \text {m}$ to highlight their relative sizes. The black mesh illustrates elastic deformations by depicting the deformation of the substrate material reference frame and highlighting the singularity at the TPCL. This mesh differs from the finer computational mesh used in simulations.

For length scales below *a*, the interfaces can be understood as diffuse and interface forces are smoothed out over corresponding distances. On length scales above *a*, we distinguish the following regimes: (i) the rigid substrate $\lambda _\textrm{c}\ll a \ll R$, (ii) the moderately soft substrate $a\ll \lambda _\textrm{c}\ll R$ and (iii) the soft limit $a\ll R\ll \lambda _\textrm{c}$, e.g. ^7^. The rigid limit (i) is solely governed by Young’s law^8^ and substrate deformations are practically invisible. The case (ii) still allows for Young’s law on the macroscopic scale, but satisfies Neumann’s law near the TPCL^9^, where an elastic ridge becomes detectable. In the soft limit (iii) elastic properties can be mainly neglected and stationary droplets are liquid lenses determined by the Neumann triangle. For a more detailed discussion, including the regimes of thin, thick, and semi-infinite substrates, we refer to^10^. Typical droplets for various radii on moderately thick substrates $H/R>1$ obtained by minimization of elastic and surface energy are shown in Fig. 1. Experimental measurements of the elastocapillary ridge for moderately soft but also thinner substrates $H=50\,\upmu \text {m}$ can be found in^11^ for $\lambda _\textrm{c}\approx 10\,\upmu \text {m}$ and droplet radii in the range $27\,\upmu \text {m}<R<226\,\upmu \text {m}$.

The Shuttleworth effect relates the surface tension to the surface energy depending on a (specific) surface area, where for one-component liquids they are assumed to be equal^1^. This relation implies that for solid surfaces, surface tension and surface energy are different concepts. While experimental evidence for a positive Shuttleworth effect for PDMS samples was presented in^11–17^ it was not observed in^18^ and argued that no Shuttleworth effect is expected in case of a polymer with reduced cross linking density at the interface. So the resulting role and interpretation of the Shuttleworth effect has been slightly controversial. Despite this controversy, it is generally accepted that force balances at a TPCL are derived using variational energetic arguments as in^19^ or^14,20^, where the former employs dimension-reduced models with disjoining pressures and the latter two show that in addition to Neumann’s law another condition is derived and ensures a *no-pinning* condition.

Beside the balance of elastic and surface energies, there are a couple of irreversible phenomena that emerge from the viscoelastic or poroelastic nature of soft substrates. It is well known that stationary droplets can generate substantial stresses that lead to crack formation (fracture) in underlying soft substrates^21^. In dynamic wetting of soft substrates at sufficiently high velocity, contact lines do not move continuously but in a series of stick-slip events, e.g.^22,23^, that are connected to the viscoelastic nature of the substrate encoded in the ratio of loss and storage modulus. Similar to Navier-slip, this effect is based on a characterization of an effective interfacial dissipation similar to Navier-slip observed in viscoelastic adhesion, where such a stick-slip process induces the rupture of adhesive bonds^24,25^ in the vicinity of a TPCL. The distinction between viscoelastic and poroelastic effects is slightly ambiguous, but^3^ argue that both are essential to fully describe the relaxation dynamics of soft polymeric gels.

Upon realizing that soft substrates can be inhomogeneous materials, such as swollen aqueous hydrogels or polymer melt networks with uncrosslinked chains that can be transported within the substrate by elasto-chemo-capillary forces^26^, it becomes evident that these transport processes can significantly alter dynamic properties. Examples include the temporal formation of a wetting ridge^27^ or the slow relaxation of a wetting ridge formed at the TPCL^3^, i.e., towards or away from regions of highest strain. These transport phenomena are particularly pronounced in strongly swollen networks, where it has been shown that uncrosslinked polymer phase-separates from the network near the TPCL, influencing the adhesive properties of the gel^28^. The same effect has been observed in static and dynamic wetting ridges of water droplets on swollen PDMS substrates^29,30^. To the best of our knowledge, a fully coupled model that integrates viscoelastic and poroelastic effects for wetting problems has yet to be developed.

In systems with multiple surface tensions $\gamma _{ij}$, stability conditions govern the existence and stability of stationary droplet configurations and of contact angle force balances. In particular for slippery surfaces, *cloaked droplets* that are completely wetted by a lubricant exist^31^. A corresponding dynamical transition to cloaked droplets has been shown theoretically using molecular dynamic simulations in^32,33^. Depending on droplets being completely or partially cloaked, this effect can modify the effective surface tensions and thereby change the observed wetting dynamics^11,17,29^; it can also lubricate the droplets^34,35^ increasing the velocity of sliding drops or reducing wetting hysteresis.

Multiple experimental techniques have been used over the years to explore the morphology of interface shapes on soft substrates, e.g., optical interferometry^2^, fluorescent confocal microscopy^36^, and X-ray microscopy^13^. Interferometry provides excellent height resolution but is restricted laterally to optical resolution and limited to contact angles of about $30 ^{\circ }$ while confocal microscopy or similar modern optical microscopy techniques provide a lateral resolution down to about 200 nm or even below with a height resolution that does not quite match the lateral resolution, which might be particularly restrictive near the TPCL. X-ray microscopy has pushed the spatial resolution to $\sim 50$ nm and enables visualization of buried interfaces without relying on optical transparency; however X-ray imaging relies on contrasts in electron density. AFM typically offers a lateral and height resolution in the sub-nanometer range and is thus superior to the resolution offered by typically used optical or X-ray methods. It enables imaging slopes up to about $60^{\circ }$ but comes with the drawback of increased data acquisition time and that liquid structures can be delicate to image. While imaging still works in a straightforward manner for liquid PS structures, as used in the present study, even at elevated temperatures, e.g.^37–39^, are aqueous structures quite demanding and might need a hygroscopic stabilization of the liquid morphologies, cf.^40,41^. AFM techniques combined with lift-off methods, *i.e.*, removal of one phase followed by measurement of the buried interface^42,43^, additionally allows imaging of buried interface.

In this study, we investigate stationary polystyrene (PS) droplets on two types of polydimethylsiloxane (PDMS) substrates (Sylgard 184 (SG184) and Sylgard 186 (SG186)) in the moderately soft regime, where the elastocapillary lengths are $\lambda _\textrm{c}\sim 30\ \text {nm}$ and $\lambda _\textrm{c}\sim 80\ \text {nm}$, respectively. The radii of the considered droplets range between $300 \ \text {nm}$ and $3 \ \upmu \text {m}$. Using Atomic Force Microscopy (AFM), we directly obtain the topographies of the PS-air and PDMS-air interfaces.

To expose the internal PS-PDMS interface, we employ a novel lift-off technique combined with AFM. We rigorously compare these experimental measurements with theoretical predictions of axisymmetric droplet shapes, based on nonlinear neo-Hookean elasticity where capillary surfaces are modeled with constant surface tension. These comparisons emphasize droplet sizes and features near the three-phase contact line (TPCL), which are challenging to resolve with conventional optical or X-ray techniques. To address observed discrepancies near the TPCL, we consider the relevance of nonlinear effects, including viscoelasticity, poroelasticity, the Shuttleworth effect, cloaking, and potential phase separation—an effect not previously identified for un-swollen PDMS substrates. While these nonlinear effects are often treated separately in the literature, they may act together to influence the observed phenomena.

## Experimental methods and theoretical models

### Experimental methods

As support for the PDMS samples, silicon wafer cuts ($\langle 100 \rangle$, $375~\upmu \text {m}$, Si-mat) about $(1 \times 1)~\text {cm}^{2}$ were cleaned by sonication in ethanol, isopropanol, and toluene, followed by a $30~\text {s}$ treatment in a plasma cleaner (Diener electronic Femto). PDMS silicone elastomer kits Sylgard 184 (SG184) and Sylgard 186 (SG186) from Dow were used and mixed according to the manufacturer’s specifications. The degassed PDMS mixtures were spin coated on the cleaned Si wafer cuts for $5~\text {min}$ at spin frequencies of $\omega = 6000~\textrm{rpm}$ for SG184 and $\omega = 8000\,\textrm{rpm}$ for SG186 to obtain uniform film thicknesses of $(7 \pm 2)~\upmu \text {m}$ (measured by AFM) and cured on a hot plate at $75~^{\circ }\text {C}$ for $90~\text {min}$.

The shear modulus of PDMS bulk samples were determined using a frequency sweep test using a Haake-Mars-40 rheometer in plate-plate geometry with a radius of $25 \ \text {mm}$. To guarantee good mechanical contact of PDMS and shear geometry, the PDMS mixtures were cured in the shear geometry at $80~^{\circ }\text {C}$ to obtain a shear modulus of $G_\textrm{SG184}=595$ kPa for Sylgard 184 and of $G_\textrm{SG186}=224$ kPa for Sylgard 186.

**Fig. 2:**
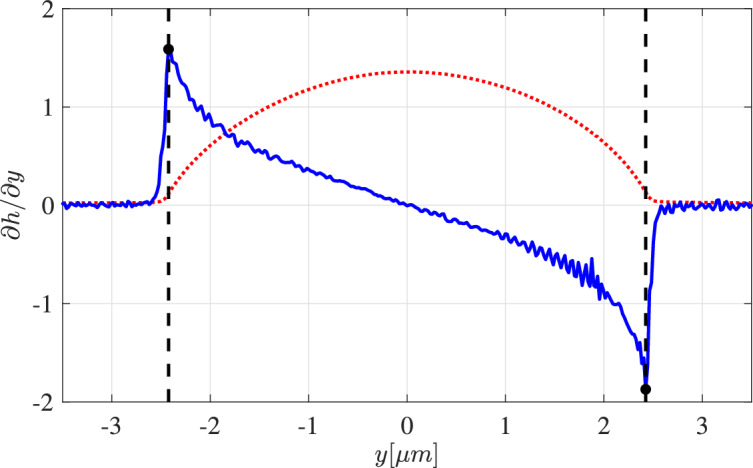
Droplet cross-section on SG184 obtained with AFM (red dots) with corresponding first derivative (blue solid). TPCL is visible from the kink in the first derivative and is marked by a black dot and the black dashed line.

Small polystyrene (PS) droplets on PDMS were prepared by dewetting. For that, PS layer with a thickness of $120~\text {nm}$ were first prepared in a glassy state by spin coating a PS-toluene solution on a freshly cleaved mica sheet. The thus prepared glassy PS layer is then floated on an ultra-pure water interface (Fisher Scientific) and picked up from there with a previously prepared PDMS coated Si substrate. The used atactic PS is a gel permeation standard, purchased from Polymer Standards Service (Mainz, Germany), with a molecular weight of $17.8~\text {kg mol}^{-1}$ and a polydispersity index of ${M_w}/{M_n} = 1.04$, where $M_w =17.4~\text {kg/mol}$ is the weight average molecular weight and $M_n=16.8~\text {kg/mol}$ is the number average molecular weight. To avoid contamination, sample preparation was done in an ISO 5 clean room atmosphere. The prepared PS/PDMS samples were annealed at $T_\textrm{a}=(120 \pm 1)~^{\circ }\text {C}$, that is about 20 K above the glass-transition temperature of PS(17.8k). During the annealing period, the initially uniform PS layer becomes liquid and transforms into droplets by dewetting from the underlying PDMS substrate. To ensure that the obtained PS droplets are in, or very close to equilibrium, the SG184 samples were typically annealed for two days and the obtained droplets seem perfectly round. Additional experiments with annealing periods of up to eight days revealed no changes after the second day and in particular no volume loss. In case of SG186 samples, the dewetting is substantially slower and annealing times of 20 days were applied. However, full equilibration exceeded experimentally accessible time scales and even after more than 20 days of annealing, the history of the dewetting pathway is still visible by a slightly elliptical footage in the horizontal plane of the droplets resulting in contact angles that vary slightly between the long and the short axis of the droplet, see Supplement SI2.

The 3D shape of the obtained equilibrium PS droplets sitting on the PDMS substrate were analyzed by atomic force microscopy (AFM, Bruker Dimension FastScan), using Olympus tips (OMCL-AC160TS-R3) in Soft Tapping Mode. Determining the droplet shapes together with the local mechanical properties using peak force QNM mode failed around the TPCL. This might be due to the pronounced topography at this spot but could also reflect a liquid rim surrounding the PS droplet below the TPCL, as discussed later. AFM scanning was typically obtained at room temperature, where the PS droplets are in a glassy state, which reduces the probability of contaminating the probes. However, test experiments were also carried out at dewetting temperatures of $T=120~^{\circ }\text {C}$, where the droplets were in the liquid state, and no difference in the droplet shape were observed. A droplet surface obtained by AFM together with the first derivative is shown in Fig. 2. Although the position of the TPCL cannot be inferred from a jump in the first derivative of the height profile, the contact line gives rise to a physically relevant signature in the second derivative. In particular, the first derivative of the height remains continuous while it has a kink, implying a jump discontinuity in the second derivative at a clearly distinguished contact line position. This position coincides with the turning point of the upper AFM scan and is robustly determined by the maximal or minimal slope, even though evaluation of the second derivative of experimental data is typically too noisy to be informative.

To additionally image the buried PS-PDMS interface of the droplets, a lift-off technique was applied. To this end, a UV-curable glue layer (Norland optical adhesive, NOA 60) was poured on top of the PDMS sample embedding the glassy PS droplets. The glue is cured at a wavelength of $\lambda = 366~\text {nm}$ (Benda UV lamp) for $15~\text {min}$. Removing the cured glue from the PDMS sample, the PS droplets remain attached to the glue and can be lifted off from the PDMS substrate, and imaged by AFM.

The shape of the formerly PS-PDMS and PDMS-air interfaces is aligned with the previously scanned PS-air and PDMS-air interfaces to construct a complete 3D shape; see also the schematic diagram in Supplement SI2.

To obtain information about the chemical composition of our sample surface, the NanoIR (Bruker) technique was used. This technique uses frequency tuned infrared laser pulses to heat the top layer of the sample that is most pronounced in case of resonance absorption. The local heating results in thermal expansion, i.e., in a mechanical response of the surface, proportional to the frequency dependent absorption coefficient. This way a relative IR spectrum between 2 and 20 micrometers can be obtained with the lateral resolution of an AFM.

### Theoretical models

In the following, we set up a model for the computation of stationary liquid droplets and viscoelastic substrates with moving capillary interfaces. Several mathematical approaches describe fluid–solid interactions with capillary interfaces. Commonly, sharp-interface models, phase-field models, or their combinations are used^44–48^. Phase-field models are attractive since they use standard numerical methods with local adaptivity and robust nonlinear solvers, whereas sharp-interface models require Lagrangian or Arbitrary Lagrangian–Eulerian techniques to capture evolving free interfaces. A main drawback of phase-field models is the introduction of artificial diffusive time scales, which demand proper mobility scaling to obtain the sharp-interface limit $\varepsilon \rightarrow 0$ for stationary solutions $T\rightarrow \infty$^47,49^. To avoid these issues, we employ sharp-interface models in this paper.

**Fig. 3:**
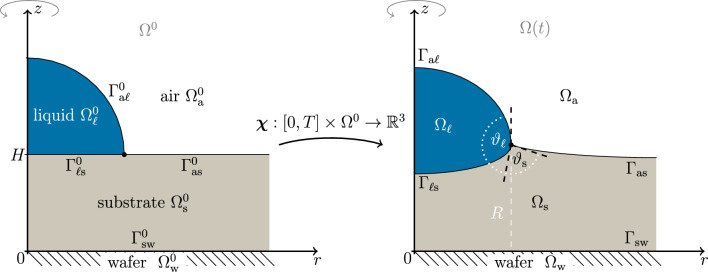
Sketch of axisymmetric liquid droplet (blue shading) on a viscoelastic substrate (gray shading) with contact line (black dot) and surrounding air phase (white) on a rigid wafer (stripe pattern) and capillary interfaces (black lines). The left side shows the reference domain (Lagrangian) $\Omega ^0$ and the right side shows the deformed configuration (Eulerian) $\Omega$ and all referential and deformed interfaces $\Gamma _{ij}(t)=\varvec{\chi }(t,\Gamma ^0_{ij})$ for $i,j\in \{\textrm{a},\ell , \textrm{s}, \textrm{w}\}$. The (deformed) droplet radius *R* is indicated by a white dashed line. We denote the solid contact angle between $\Gamma _\textrm{as}$ and $\Gamma _{\ell \textrm{s}}$ by $\vartheta _\textrm{s}$ and the liquid contact angle between $\Gamma _{\ell \textrm{s}}$ and $\Gamma _\mathrm{a\ell }$ by $\vartheta _\ell$. The substrate is initially flat $\Omega ^0_\textrm{s}=\{\varvec{x}\in \mathbb {R}^3:0\le z\le 1\}$ and the liquid droplet is the half of an ellipsoid $\Omega ^0_\ell =\{\varvec{x}\in \mathbb {R}^3:\nicefrac {r^2}{r_x^2} + \nicefrac {(z-H)^2}{r_z^2}\le 1 \text { and }z\ge H\}$ with $\varvec{x}=(x,y,z)$ and $r^2=x^2+y^2$. The substrate is supported by a rigid wafer $\Omega ^0_\textrm{w}=\{\varvec{x}\in \mathbb {R}^3:z\le 0\}$ surrounded by an ambient air domain $\Omega ^0_\textrm{a}=\mathbb {R}^3\setminus (\Omega _\textrm{s}\cup \Omega _\textrm{w}\cup \Omega _\ell )$.

With the goal to describe a simplified relaxation into a stationary state, we will use a sharp-interface model that features an initially flat substrate $\Omega ^0_\textrm{s}$ and a liquid droplet $\Omega ^0_{\ell }$ which is the half of an ellipsoid. The viscoelastic substrate is supported by a rigid wafer $\Omega ^0_\textrm{w}$ surrounded by an ambient air domain $\Omega ^0_\textrm{a}=\mathbb {R}^3\setminus (\Omega _\textrm{s}\cup \Omega _\textrm{w}\cup \Omega _\ell )$ as explained in Fig. 3. Between different domains we define interfaces $\Gamma ^0_{ij}={\Omega }^0_i\cap {\Omega }^0_j$ for $i,j\in \{\mathrm{a,\ell ,s,w}\}$. In particular, the initial undeformed substrate $\Omega ^0_\textrm{s}$ is a reference domain of stress-free elastic material.

On the (axisymmetric computational) domain $\Omega ^0=\Omega ^0_\textrm{s}\cup \Omega ^0_\ell$ we define the deformation $\varvec{\chi }:[0,T]\times \Omega ^0\rightarrow \mathbb {R}^3$, which maps the initial (referential) solid and the liquid domain to the time-dependent domains $\Omega _i(t)=\varvec{\chi }(t,\Omega ^0_{i})$ for $i\in \{\text {s},\ell \}$ and correspondingly the interfaces to the time-dependent capillary interfaces $\Gamma _{ij}(t)=\varvec{\chi }(t,\Gamma ^0_{ij})$ for $ij\in \{\ell \textrm{s}, \textrm{as}, \textrm{a}\ell \}$ with the goal to determine the stationary shapes as $t\rightarrow \infty$. Throughout the paper we denote the deformation gradient by $\varvec{F}:=\nabla \varvec{\chi }$. Here and below, we use boldface for vector and tensor quantities.

With the given geometrical definitions, the cornerstone of the sharp-interface model is the free energy $\mathcal {F}$, which for a given deformation $\varvec{\chi }$ measures the elastic energy and the surface energy. It is defined as

2 $$\begin{aligned} \mathcal {F}(\varvec{\chi }) =&\int _{\Omega ^0_\textrm{s}}\!W_{\text {elast}}(\varvec{F})\,\textrm{d}V + \int _{\Omega ^0}\!\tfrac{K}{2}(\det \varvec{F}-1)^2\,\textrm{d}V \nonumber \\&+ \sum _{ij\in \{\ell \mathrm{s,\,as,\,a}\ell \}}\int _{\Gamma ^0_{ij}} W^{\Gamma }_{ij}(\varvec{F},\varvec{\nu })\,\textrm{d}S\,, \end{aligned}$$

where $\varvec{\nu }$ is a normal vector field on respective interface $\Gamma _{ij}$ and *K* is the bulk modulus of the materials. We assume that the elastic energy density $W_{\text {elast}}$ is of neo-Hookean type and the surface/interface energy density $W^{\Gamma }_{ij}$ measures the deformed interface length multiplied with the surface tension coefficient, which gives rise to

3a $$\begin{aligned}&W_{\text {elast}}(\varvec{F}) = \frac{G}{2}\textrm{tr}(\varvec{F}^T\varvec{F}-\varvec{I}), \end{aligned}$$

3b $$\begin{aligned}&W^{\Gamma }_{ij}(\varvec{F},\varvec{\nu }) = \gamma _{ij}|\textrm{cof}(\varvec{F})\cdot \varvec{\nu }|. \end{aligned}$$

Here, *G* is the shear modulus of the substrate, $\varvec{I}\in \mathbb {R}^{3\times 3}$ the identity matrix and we denote $\textrm{cof}(\varvec{F})=\det (\varvec{F})\varvec{F}^{-1}$ the cofactor matrix of $\varvec{F}\in \mathbb {R}^{3\times 3}$. Upon non-dimensionalization of the free energy, the elastocapillary length (1) appears as a key parameter of the system. We summarize the main physical assumptions that are used in this model:

i. At the substrate-liquid interface we assume a no-slip boundary condition, i.e. $\varvec{\chi }(t,\varvec{x})$ is continuous at $\varvec{x}\in \Gamma ^0_{\ell \textrm{s}}$. On the substrate-wafer interface, we have a no-slip condition $\varvec{\chi }(t,\varvec{x})=\varvec{x}$ for $\varvec{x}\in \Gamma ^0_\textrm{sw}$.
ii. The viscoelastic substrate is hyperelastic, i.e. the deformation in equilibrium is determined by the minimization of an elastic energy $W_\textrm{elast}$ that depends on the deformation gradient $\varvec{F}$. In particular, we do not consider inelastic deformations.
iii. The substrate and the liquid are both nearly incompressible, *i.e.*, $K\gg G$.
iv. The liquid is Newtonian, and diffusive effects in the substrate are neglected. The adoption of the $W^{\Gamma }_{ij}$ in (3b) with constant surface tensions $\gamma _{ij}$ results in a spatially uniform Eulerian surface energy density on each $\Gamma _{ij}$, thereby excluding the Shuttleworth effect. Nevertheless, when deformation gradient dependence is considered and adequate material data is available, exploring more complex forms of the surface energy as described in (3b) becomes feasible and relevant, e.g. cf.^50^.

The main goal of our theoretical considerations is to compute equilibrium states, which minimize the free energy (2). Therefore, we use a simple but robust transient model that solves for $\varvec{\chi }(t)$ as $t\rightarrow \infty$. Motivated by the energetical structure of the model with the free energy (2), we use a dynamical model for the evolution that satisfies the weak form

4 $$\begin{aligned} \mathcal {D}(\partial _t \varvec{\chi }, \varvec{v}) :=\sum _{i\in \{s,\,\ell \}}\int _{\Omega _i} \mu _i\, \nabla \partial _t\varvec{\chi }:\nabla \varvec{v}\,\textrm{d}V= -\langle \textrm{D}\mathcal {F}(\varvec{\chi }),\varvec{v}\rangle \,, \end{aligned}$$

for all suitable test velocity vector fields $\varvec{v}$ with given initial values $\varvec{\chi }(t=0)$. In (4), the bilinear form $\mathcal {D}$ encodes the energy dissipated by the flow with velocity $\partial _t \varvec{\chi }$ and we used the directional derivative

$\langle \textrm{D}\mathcal {F}(\varvec{\chi }),\varvec{v}\rangle =\lim _{h\rightarrow 0} \tfrac{1}{h}\big (\mathcal {F}(\varvec{\chi }+h\varvec{v})-\mathcal {F}(\varvec{\chi })\big )$ to compute mechanical bulk and surface forces consistently. To facilitate convergence to a stationary state, we use a Kelvin-Voigt-type dissipation with constant viscosities $\mu _i$ for the solid and liquid phases $i\in \{\textrm{s},\ell \}$, taking $\mu _i = \mu \in \mathbb {R}$ for simplicity. By testing (4) with $\varvec{v}=\partial _t \varvec{\chi }$ and using the functional chain rule $\langle \textrm{D}\mathcal {F}(\varvec{\chi }),\partial _t\varvec{\chi }\rangle =\tfrac{\textrm{d}}{\textrm{d}t}\mathcal {F}(\varvec{\chi })$, we obtain thermodynamic consistency

5 $$\begin{aligned} \frac{\textrm{d}}{\textrm{d}t}\mathcal {F}\bigl (\varvec{\chi }(t)\bigr ) = - \mathcal {D}(\partial _t\varvec{\chi },\partial _t\varvec{\chi })\le 0. \end{aligned}$$

For a dynamic model moving contact lines that are not pinned, the assumption i.) presents a serious restriction. We overcome this restriction for axisymmetric minimizers by choosing the initial ellipsoid shape, i.e., $r_x$ and $r_z$, such that for a given drop volume the stationary free energy is minimized, thereby satisfying the third *no-pinning* boundary condition discussed in^14,20^. In Supplement SI4 we provide additional details on the spatial and temporal discretization of (4), implemented using the FEniCS finite element library^51^.

## Stationary droplet shapes

**Fig. 4:**
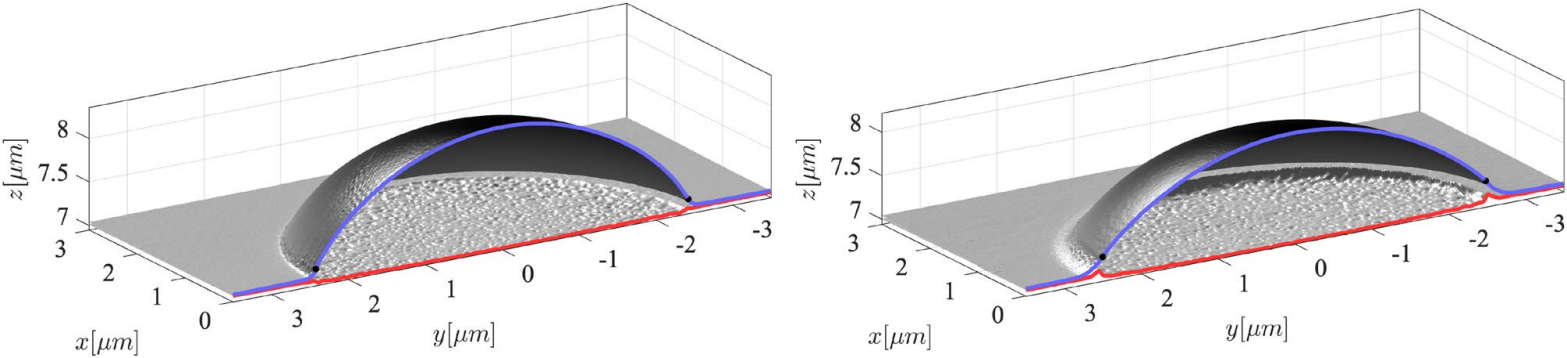
3D cross sections of top and bottom AFM scans of PS droplets with radius $R\approx 2.5\,\upmu \text {m}$ on SG184 (left) and SG186 (right); substrate height is $H\approx 7\,\upmu \text {m}$. Blue and red lines show the central top and bottom AFM scan lines, whereas the black dots denote the positions of the contact line identified by the inflection points of the top scan line.

Three-dimensional representations of experimentally obtained equilibrium droplet shapes on SG184 and SG186 substrates are shown in Fig. 4 for the largest sets of droplet radii. These configurations are characterized by $R \approx 2.5\,\upmu \text {m}$, $\lambda _\textrm{c}= \gamma _\textrm{as}/ G \sim 30\,\text {nm}$ for SG184, and $\lambda _\textrm{c}= \gamma _\textrm{as}/ G \sim 80\,\text {nm}$ for SG186, corresponding to the regime of a *moderately soft substrate*, where $a \ll \lambda _\textrm{c}\ll R$ and the substrate thickness $H=7\,\upmu \text {m}$ is larger than the droplet radius but not in the semi-infinite substrate thickness regime.

We note that within a dewetting time of several hours at $T_\textrm{a}=(120 \pm 1)\,^{\circ }\text {C}$, droplets on SG184 always reached a rotationally symmetric shape, while the equilibration of droplets on SG186 was not fully completed and the droplet bases remained slightly elliptical even after several days. The corresponding largest and smallest radii for PS droplets on SG184 vary by $3\,\%$, i.e. within the accuracy of the AFM, while the drop radii on SG186 vary by about $13\,\%$, see the 3D images and cross sections in Supplement SI2. For the large droplets with $R\gg \lambda _\textrm{c}$ shown in Fig. 4, a spherical arc fit can be used to determine effective Young angles, see also Supplement SI2.

The PS-PDMS interface of the droplet with the elastic substrate is deformed downwards by the Laplace-pressure inside the droplet. This deformation is relatively small because the elasto-capillary length $\lambda _\textrm{c}$ is small compared to the drop radius *R*. We also note that all AFM scans of the PS-PDMS interface show some roughness in comparison to the PS-air and PDMS-air interface, which may indicate low PS-PDMS interfacial tension but could also be due to aggregation of filler particles. This roughness is characteristic for the respective substrate and independent on drop size.

The experimental AFM profiles of the top surface transition smoothly from the PS-air interface to the PDMS-air interface and, in particular, do not show any kink at the TPCL. As explained in the Methods section, the TPCL is determined and indicated by the black dot on top of the blue scan line in Fig. 4. The TPCL is pulled upwards with respect to the undeformed PDMS substrate to accommodate the forces generated by the surface tensions $\gamma _{ij}$ for $i,j\in \{a,s,\ell \}$ for the interfaces between the air (*a*), substrate (*s*) and liquid ($\ell$) phases. The balance of interfacial forces leads to a Neumann triangle, that generates the characteristic elastic ridge below the TPCL^10^. Furthermore, note that the shown top contours align with the bottom contour of the soft substrate at a slight distance from the droplet and the TPCL. However, in the immediate vicinity of the TPCL shown in Fig. 4, the upper and lower AFM profiles do not align, creating the impression that a small volume of PDMS is missing. This missing alignment near the TPCL is present in all experiments and is enhanced for the softer SG186 substrate.

**Table 1:** Interfacial tensions at $T_\textrm{a}=120^{\circ }\text {C}$ from literature (left column) and from hybrid construction (6) for large cloaked droplets (right column) for SG184 and SG 186. Values in the right column for SG186 are based on Young angle along the shorter/longer axis; the errors in parenthesis additionally represent the uncertainty of the reference value, $\gamma _\textrm{as}$ for SG184 and SG186.

Interface	Surface tension $[\text {mN m}^{-1}]$	Surface tension $[\text {mN m}^{-1}]$	
	Literature	SG184	SG186
PDMS-air, $\gamma _\textrm{as}$	$15 \pm 1$ ^52,53^	$15\,(\pm 1)$	$15\,(\pm 1)$
PS-air, $\gamma _{\textrm{a}\ell }$ / $\gamma _{\textrm{a}\ell }^c$	$31\pm 1$ ^42,54,55^	$19.2 \pm 0.1\,(\pm 1.3)$	$18.8/17.8\,(\pm 1.2)$
PS-PDMS, $\gamma _{\ell \textrm{s}}$	$0-10$ ^56^	$4.2 \pm 0.1 \,(\pm 0.3)$	$3.8/2.8\,(\pm 0.2)$

For equilibrium droplet shapes, the relevant system parameters are the interfacial tensions of PS-PDMS, PS-air, and PDMS-air and the shear modulus *G* of PDMS. In the middle column of Table 1 we list the literature values for the corresponding interfacial tensions, which are not compatible with an equilibrium Neumann construction since the stability condition $\gamma _\textrm{as}+\gamma _{\ell \textrm{s}}\ge \gamma _{\textrm{a}\ell }$ is violated, cf. Fig. 3. This means that, based on these literature values, stable PS droplet configurations on PDMS that adhere to the Neumann construction are impossible, which is in clear contrast to our experimental observations shown in Fig. 4.

**Fig. 5:**
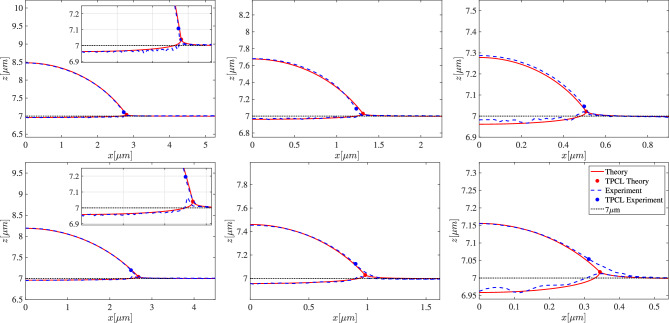
Comparison of experimental AFM cross section (blue dashed line) for Sylgard 184 (top) and Sylgard 186 (bottom) and theoretical prediction (red line) for different droplet radii decreasing from left to right. The position of the TPCL is shown using a dot and the inset shows a close-up of the liquid-substrate interface. The horizontal dotted line shows the undeformed PS-PDMS/undeformed PDMS-air interface assumed at exactly $z=7\,\upmu \text {m}$. The slight roughness of the PS-PDMS interface has an amplitude $\pm 10$ nm independent on the drop size and becomes more visible on a smaller (relative) droplet scale.

Using NanoIR (Bruker), we observed a layer of liquid PDMS along the PS-air interface, a phenomenon known as cloaking^32,57^. The observed strength of the PDMS signal suggests that the thickness of the cloaking layer is thick enough to lower the surface tension of the bare PS-air interface, $\gamma _{\textrm{a}\ell }= (31\pm 1)~\text {mN m}^{-1}$, throughout the droplet-air interface^42,54,55^ to that of a fully cloaked PS-air interface, $\gamma _{\textrm{a}\ell }^{c}$, by

6a $$\begin{aligned} \gamma _{\textrm{a}\ell }^{c} = \gamma _{\ell \textrm{s}}+\gamma _\textrm{as}. \end{aligned}$$

This situation of a fully cloaked droplet is equivalent to a degenerate Neumann construction with $\vartheta _\textrm{s}=0$ and $\vartheta _\ell =180^{\circ }$, see Fig. 3, which corresponds to the experimentally observed smooth transition from the PDMS/air interface to the cloaked PS/air interface visible in Fig. 4, thereby providing the physical explanation for the absence of a discontinuity in the first derivative of the height and its appearance in the second derivative. However, since the effective surface tension of the cloaked PS surface, $\gamma _{\textrm{a}\ell }^{c}$ directly depends on $\gamma _{\ell \textrm{s}}$, which varies with the chain length and temperature of PS and PDMS, cf. Table 1 and^56^, literature values, provide only a relatively broad range for the surface tension at typical dewetting temperatures of $T_\textrm{a} = (120 \pm 1)^{\circ }\text {C}$. With the help of a hybrid construction that applies the effective Young contact angle $\theta$ to a horizontal substrate, the PS-PDMS interfacial tension $\gamma _{\ell \textrm{s}}$, can be determined specifically for our system based on experimental observations. This construction is well-defined for $R \gg \lambda _\textrm{c}$, i.e., for large droplets where the deformation of the PDMS substrate is relatively small compared to $\lambda _\textrm{c}$, see e.g.^10^. Using Young’s equation

6b $$\begin{aligned} \gamma _{\ell \textrm{s}}= \gamma _\textrm{as}- \gamma _{\textrm{a}\ell }^c\cos (\theta ), \end{aligned}$$

and inserting (6a), $\gamma _{\ell \textrm{s}}$ can be calculated by reconstructing the effective Young’s angle $\theta$ by fitting spherical arcs to radial cross sections of the experimental droplet shapes. For SG184, this yields $\theta = (55.9 \pm 0.3) ^{\circ }$, with the error estimated by comparing spherical arcs fitted along the longest and shortest radial cross-section of the measured droplet profiles. We note that for PS droplets on SG186, we observe stronger deviations from an axisymmetric droplet shape, so that the contact angle for the largest droplets varies between $\theta =52.7^{\circ }$ (short axis) and $\theta =47.0^{\circ }$ (long axis). Corresponding AFM images and cross-sections can be found in Supplement SI2 together with a discussion on how to estimate contact angle hysteresis and why possible effects from pre-strain can be excluded. The resulting PS-PDMS surface tension obtained by this hybrid construction is around $\gamma _{\ell \textrm{s}}\approx 5 ~\text {mN m}^{-1}$. Inserting the corresponding value for $\gamma _{\ell \textrm{s}}$ in (6a) finally leads to the surface tension of a fully cloaked PS surface of $\gamma _{\textrm{a}\ell }^{c} \approx 19 ~\text {mN m}^{-1}$. The precise values for SG184 and SG186 along short and long axis are provided in the right column of Table 1 and were used as input parameter to computed droplet shapes.

To quantitatively match numerical and experimental droplet shapes, the three-dimensional volume of each experimental droplet was computed under the assumption of axisymmetry of the cross-section along a given scan direction, resulting in the volumes provided in the table in Supplement SI4. These volumes were converted into radii $r_x, r_z$ used for initial data described in Fig. 3. Accordingly, for a given shear modulus *G* and interfacial tensions $\gamma _{ij}$, stationary solutions were computed solving (4) leading the energy minimizers of (2). For the large droplets $R=(2-3)\,\upmu \text {m}$ with $\lambda _\textrm{c}\ll R$, the PS-PDMS interface reveals a small but in the close up clearly visible depression below the droplet, Fig. 4. Cross sections of experimentally measured depressions of the largest droplets are used to fit the shear modulus *G* of the PDMS substrate that provides the best match between numerical and experimental data. For SG184, the thus obtained shear modulus is $50\%$ lower than the experimentally measured one, $G_\textrm{SG184}=298\,\text {kPa}=0.5 \, G_\textrm{SG184}^\text {exp}$. For SG186, we find that the fitted value agrees well with the experimentally determined value, $G_\textrm{SG186}=G_\textrm{SG186}^\text {exp}=224\,\text {kPa}$. A reduction of the shear modulus can be rationalized by a nonuniform crosslinking density in PDMS films. In particular, a reduced crosslinking density is expected near the PDMS-air interface^34,58^, explaining the reduced shear modulus for SG184 obtained from fitting the numerical data compared to bulk rheology measurements. From this perspective, it is rather surprising that the bulk value $G_\textrm{SG186}$ for SG186 can be used without further correction, which might be a result from the not fully equilibrated PS drop shapes on SG186. However, the shear moduli for SG184 and SG186 obtained from fitting large droplets are then used to compute stationary states of smaller droplets without any further adjustments.

With the determined experimental droplet volumes provided in Supplement SI4, the hybrid construction to determine surface tensions and the adjusted shear moduli, we have derived theoretical stationary droplet configurations that can be compared with the corresponding experimental results as shown in Fig. 5. Droplet shapes computed for different volumes on SG184 and SG186 substrates are shown by red solid lines in Fig. 5 together with the corresponding experimental droplet shapes (blue dashed lines). Corresponding experimental and theoretical TPCL positions are shown with the same color coding using solid dots. For the largest droplet radii on SG184 and SG186, the inset highlights that matching indentation of the PS-PDMS interface.

All shown configurations on SG184 and SG186 show excellent overall agreement for the determined interface shapes for the PS-air, PS-PDMS and PDMS-air interface based on classical elastocapillarity. Within the limits given by the interfacial roughness, the indentation of the PS-PDMS is well captured and the constant curvature of the PS-air interface perfectly matches the theoretical prediction of the axisymmetric theoretical profiles. While large droplets $R>1\,\upmu \text {m}$, shown in the left and middle panel of Fig. 5, look qualitatively similar, the smaller droplets with $R<0.5\,\upmu \text {m}$ in the right panel show the transition from moderately soft substrates towards the soft limit in terms of stronger relative deformation of the PS-PDMS interface. Droplets with $R \ll \lambda _{\text {cap}}$, much smaller than those probed experimentally, assume equilibrium shapes similar to the liquid lenses on liquid substrates^42^, e.g. see right panel of Fig. 1.

Finally, we note that the softer SG186 generally shows larger deformations of the PS-PDMS interface for similarly-sized droplets. One general difference between experimental and theoretical interface shapes is that, although the theoretical prediction employs a degenerate Neumann triangle construction (6a), the transition from the PS-air to the PDMS-air interface does not appear as smooth as in the experiments on the scale of the droplet radius.

In the following, we will use the general good agreement of the global experimental and theoretical droplet shapes and focus on the discrepancies near the TPCL. While the trends and interface shapes for different droplet sizes agree well with the experimental results for all substrates, we observe a systematic deviation in the immediate vicinity of the TPCL, which cannot be explained by classical elastocapillarity when applying realistic physical parameters. This trend enhances for decreasing droplet size and for softer substrates, see Fig. 5. The exact shape of the PS-PDMS interface near the contact line appears difficult to predict due to the missing alignment of the top and bottom AFM scan at the TPCL, see Figs. 4 and 5. However, already from the PS-air interface, it is clear that the experimentally measured elevation of the TPCL is raised about three times higher above the unperturbed PDMS surface than in the theoretical prediction; see dots in Fig. 5. In addition, the position of the TPCL in the experimental drop profiles is not only shifted upwards compared to the numerical drop profiles but also inwards, towards the center of the drop. Both the relative magnitude of the upward deformation and the inward shift of the TPCL, i.e., the deviation between numerical and experimental results shown in Fig. 5, increase for smaller droplets and for softer substrates with droplet sizes closer to the elastocapillary length.

To check for the origin of the observed discrepancies on the position of the TPCL between experiment and classical elastocapillary theory, we conducted several test that are described in the following. To investigate if differences in the substrate thickness and thus in the effective substrate elasticity could cause this effect, we varied the substrate thickness for the computed drop shapes in the range of $H=5-10\,\upmu \text {m}$, see Supplement SI6, and found only a small impact on the droplet shape and indentation and a negligible impact on the droplet shape near the TPCL, as long as $\lambda _\textrm{c}<H$ and $R < H$, which is satisfied for all our experimental results. Computed PS droplet shapes on SG186 for a drastically reduced shear modulus of $\tfrac{1}{16} G_\textrm{SG186}^\text {exp}$ match the elevation of the three-phase contact line, see Supplement SI8. However, then the global drop shape fails to describe the experimental profiles, indicating that the overall substrate is too soft.

Theoretical calculations with different surface tensions corresponding to different solid opening angles $\vartheta _s=0^{\circ }$ and $\vartheta _s=40^{\circ }$ but with fixed Young angle, showed no differences in the observed global shapes or in the elevation of the TPCL on the drop scale *R* (see Supplement SI5 in Fig. S9). This is consistent with the observations of Masurel et al. ^59^ and Pandey et al. ^20^, who respectively challenged or confirmed the Neumann triangle construction depending on the spatial resolution of the TPCL in their theory. Experimental verification of the actual solid opening angles $\vartheta _s$ for our PS/PDMS system would require much higher spatial resolution at the molecular scale, on the order of $a \sim 10^{-9}\text {m}$, or an adaptive meshing strategy to capture the singular nature of the capillary ridge for $\vartheta _s = 0$ in the theory. Accordingly, although highly relevant on the micro- and nanoscale and debated in the literature, variations in surface tension that alter $\vartheta _s$ do not affect ridge shape at the droplet scale and therefore are also unable to explain the experimentally observed increase in TPCL elevation.

Thus, we can conclude that, under the aforementioned physical modeling assumptions, the theoretical model has no remaining parameters to reconcile the differences near the TPCL between experimental and numerical drop shapes while maintaining the good global agreement of the predicted droplet shapes.

## Discussion and outlook

In summary, we have made the following experimental and theoretical observations: Firstly, polystyrene droplets are cloaked, which is theoretically expected as otherwise surface tension values would be incompatible with a Neumann triangle construction for stationary droplets. Secondly, in contrast to PS droplets on SG184, remain PS droplets on SG186 slightly elliptical, even after rather long experimental times. This unexpected observation could be attributed to viscoelastic effects in the crosslinked polymer network, such as stress relaxation through reversible or irreversible crosslink reformation. While a clear distinction between viscoelastic and poroelastic effects is challenging, stick-slip dynamics and hysteresis have been often linked to viscoelastic behavior in the literature^7,23^. Specifically, these effects are frequently associated with rate-dependent dissipation, where the rheology is characterized by a power-law model^23^ of the form $\mu = G(1 + (i\tau \omega )^n)$, with $n = 0.55$. This dissipation mechanism is expected to vanish in the stationary limit as $\omega \rightarrow 0$. However, the persistence of deviations from axisymmetric droplet shapes hints at a possible viscoelastic stick-slip mechanism, potentially caused by either bulk viscoelasticity or viscoelastic adhesion at the interface. And lastly, within the theoretical model class, we find good agreement between global theoretical and experimental droplet shapes with distinct differences near the TPCL, i.e. the elevation of the TPCL is systematically underestimated by the theory compared to the experimental measurements. As global parameters are unable to account for the observed mismatch between the theoretical model and the experimental results near the TPCL, localized phenomena caused by an increased elastocapillary length, $\lambda _\textrm{c}$ are expected to play a role. Such an increase could arise from a local reduction in shear modulus *G* or a local enhancement of surface tension $\gamma _{ij}$ caused by different complex mechanisms that we discuss in the following.

A locally higher surface stress could be attributed to the Shuttleworth effect^1^, which describes a strain-dependent surface stress in (soft) solids. However, the existence and significance of the Shuttleworth effect for PDMS substrates remain a topic of debate in the literature. While the majority of studies report a positive Shuttleworth effect, i.e., a stiffening of stretched interfaces both at the PDMS-air and PDMS-PS interfaces^11,12,14–17,59^, which was even quantified to increase with a slope of about $1\,\text {mN m}^{-1}$ for each percent of strain^12,60^, others suggest an asymmetric Shuttleworth effect^13^ or no observable Shuttleworth effect^18^. The latter argue that such an effect is unlikely in polymers with a reduced crosslinking density at the interface^18^, a scenario expected for our PDMS surfaces. However, to the best of our knowledge, there is no evidence in the literature supporting the existence of a negative Shuttleworth effect for PDMS substrates.

The overall stretching of the PDMS in our case is less than $1\,\%$ (vertical height of the TPCL $< 100\,\text {nm}$ at a PDMS thickness of $\sim 7\,\upmu \text {m}$) and a Shuttleworth effect based on a rather global (pre-) strain would account for an increase of the surface strain of less than $1\,\text {mN m}^{-1}$^12,60^ and can therefore be ignored. In contrast, the local surface elongation is higher and in the order $10-100\%$, but limited to a distance of about $<30\,\text {nm}$ around the TPCL, see Supplement SI7. So despite the expected Shuttleworth effect on a very local scale, its lateral extension seems way too small to account for the observed discrepancies near the TPCL. However, if the above reasoning were incorrect and a positive Shuttleworth effect were incorporated into the theory, the additional surface stiffening at the PS-PDMS and PDMS-air interfaces would shift the position of the computed TPCL even further downward, thereby increasing the observed vertical discrepancy between the numerical and experimental results. However, it has been shown in^61^ that an asymmetric Shuttleworth effect can significantly alter the contour of the PDMS surface and could hence help to match the PDMS contour. But it was also shown in^61^ that an asymmetric Shuttleworth effect has only minimal impact on the horizontal position of the three-phase contact line and thus fails to explain the discrepancy in this respect. In summary, assuming an asymmetric and negative Shuttleworth effect, one could possibly explain the observed contour of the PDMS surface, but not the position of the TPCL. And as no evidence in literature can be found for a negative Shuttleworth effect for these type of substrates and only a hint towards a possible asymmetric Shuttleworth effect^13^, which can also not fully explain the findings, it seems very unlikely that including the Shuttleworth effect in the theoretical model would explain or reduce the differences to the experiment.

What is also discussed in the context of polydimethylsiloxane substrates is poroelasticity to account for certain dynamic or static drop features^3,11,17,27–30,34,35,61^. Poroelastic effects can locally soften and swell the substrate, i.e. the shear modulus can depend on the fraction of uncrosslinked molecules *c* and concentration is coupled to the local volume through a contribution that encodes the swelling of soft gels. Corresponding theoretical models describe how elastic deformation and diffusion are coupled through mechanochemical forces, e.g.^27,62^. This coupling generates gradients in the chemical potential that drive the diffusion of *c*, leading to a slightly higher concentration of solvent or polymer molecules near the TPCL in weakly coupled systems. In contrast, a strong elasto-chemo-capillary coupling can even lead to localized phase separation or demixing in this region. Besides altered elastic properties, poroeleasticity adds a time scale to the system as shown in^3,27^ where the dynamic formation and relaxation of a wetting ridge is explored assuming poroelasticity and not just viscoelasticity as done earlier^2^.

The potential relevance of poroelasticity and of uncrosslinked molecules in a PDMS elastomers in general can be estimated from their volume fraction. While SG184 contains 4-5 %^35,63^ of uncrosslinked molecules and SG186 contains $6-7\,\%$^64^, can this fraction increase up to 60 % for certain PDMS mixtures^29^ and even more for swollen PDMS^28,29,62^. For colloids^28^ and droplets^29^ in contact with strongly swollen PDMS, a volume of demixed PDMS oligomers was observed near the TPCL establishing a smooth contact of PDMS and colloid while avoiding the otherwise occurring stress singularities at the TPCL^28,29^. In^29^ it was even speculated that the phase separation occurs for any swelling ratio but could not be detected due to technical limitations. This is in line with the well known feature that PDMS substrates can restore their hydrophilic properties after oxidation and that a reduced surface tension was observed for water or glycerin droplets that were placed on native PDMS surfaces, which can be interpreted as a thin layer of PDMS oligomers that were extracted from the bulk PDMS and that are cloaking^11,17,29^ or lubricating^34,35^ the drops. Interestingly, even in articles that neither observed cloaking or mention lubrication explicitly, it was frequently mentioned that water or glycerol drops on PDMS show no or very little hysteresis effects when given sufficient time to relax^11,12,14,16,28^, which is at least consistent with a lubrication layer, and based on surface energies also cloaking is expected in such a case. In^32^, using coarse-grained molecular dynamics, the experimentally observed cloaking was theoretically reproduced. In the future, similar continuum models describing cloaking dynamics and integrating them with established concepts such as complete wetting may provide a missing explanation for this observation in systems governed by elastocapillarity.

We therefore conjecture that the demixing observed optically near the TPCL for water droplets on strongly swollen PDMS substrates^29^ can also occur for PS droplets on native PDMS substrates, at the length scale where diffusive and elastocapillary effects are balanced. This phenomenon is able to explain the locally increased elastocapillary length, potentially accounting for both the enhanced elevation of the TPCL and the smoother transition of the (cloaked) PS-air interface to the PDMS-air interface observed in experiments compared to theoretical predictions. The hypotheses of poroelasticity and viscoelasticity require further experimental and theoretical investigation to clarify their roles and, if possible, attempt a disambiguation of their respective contributions.

## Supplementary Information

Supplementary Information.

## Data Availability

Data will be made available on request.

## References

[1] Shuttleworth, R. The surface tension of solids. *Proc. Phys. Soc. Soc. A* **63**, 444 (1950).

[2] Carré, A., Gastel, J.-C. & Shanahan, M. E. R. Viscoelastic effects in the spreading of liquids. *Nature* **379**, 432–434 (1996).

[3] Qin, X., Wilen, L. A., Jensen, K. E., Style, R. W. & Dufresne, E. R. Viscoelastic and poroelastic relaxations of soft solid surfaces. *Phys. Rev. Lett.* **125**(23), 238002 (2020). 33337191 10.1103/PhysRevLett.125.238002

[4] Style, R. W., Jagota, A., Hui, C.-Y. & Dufresne, E. R. Elastocapillarity: Surface tension and the mechanics of soft solids. *Annu. Rev. Condens. Matter Phys.* **8**, 99–118 (2017).

[5] Bico, J., Reyssat, É. & Roman, B. Elastocapillarity: When surface tension deforms elastic solids. *Annu. Rev. Fluid Mech.* **50**, 629–659 (2018).

[6] Chen, L. et al. Static and dynamic wetting of soft substrates. *Curr. Opin. Colloid Interface Sci.* **36**, 46–57 (2018).

[7] Andreotti, B. & Snoeijer, J. H. Statics and dynamics of soft wetting. *Annu. Rev. Fluid Mech.* **52**, 285–308 (2020).

[8] Young, T. An essay on the cohesion of fluids. *Philos. Trans. R. Soc. Lond.* **95**(1), 65–87 (1805).

[9] Neumann, F. *Vorlesungen über mathematische Physik: Vorlesungen über die Theorie der Capillarität*, volume 7. BG Teubner (1894).

[10] Style, R. W. & Dufresne, E. R. Static wetting on deformable substrates, from liquids to soft solids. *Soft Matter* **8**(27), 7177–7184 (2012).

[11] Style, R. W. et al. Universal deformation of soft substrates near a contact line and the direct measurement of solid surface stresses. *Phys. Rev. Lett.* **110**(6), 066103 (2013). 23432280 10.1103/PhysRevLett.110.066103

[12] Xu, Q. et al. Direct measurement of strain-dependent solid surface stress. *Nat. Commun.* **8**, 555 (2017). 28916752 10.1038/s41467-017-00636-y PMC5601460

[13] Park, S. J. et al. Visualization of asymmetric wetting ridges on soft solids with x-ray microscopy. *Nat. Commun.* **5**(1), 4369 (2014). 25007777 10.1038/ncomms5369 PMC4104447

[14] Snoeijer, J. H., Rolley, E. & Andreotti, B. Paradox of contact angle selection on stretched soft solids. *Phys. Rev. Lett.* **121**(6), 068003 (2018). 30141666 10.1103/PhysRevLett.121.068003

[15] Bain, N. et al. Surface tension and the strain-dependent topography of soft solids. *Phys. Rev. Lett.* **127**(20), 208001 (2021). 34860052 10.1103/PhysRevLett.127.208001

[16] Heyden, S., Bain, N., Qin, X., Style, R. W. & Dufresne, E. R. Contact lines on stretched soft solids: modelling anisotropic surface stresses. *Proc. R. Soc. A* **477**(2245), 20200673 (2021).

[17] Zhao, W., Zhou, J., Haitao, H., Chang, X. & Qin, X. The role of crosslinking density in surface stress and surface energy of soft solids. *Soft Matter* **18**(3), 507–513 (2022). 34919111 10.1039/d1sm01600h

[18] Schulman, R. D., Trejo, M., Salez, T., Raphaël, E. & Dalnoki-Veress, K. Surface energy of strained amorphous solids. *Nat. Commun.* **9**(1), 982 (2018). 29515162 10.1038/s41467-018-03346-1 PMC5841398

[19] Henkel, C., Snoeijer, J. H. & Thiele, U. Gradient-dynamics model for liquid drops on elastic substrates. *Soft Matter* **17**(45), 10359–10375 (2021). 34747426 10.1039/d1sm01032h

[20] Pandey, A. et al. Singular nature of the elastocapillary ridge. *Phys. Rev. X* **10**(3), 031067 (2020).

[21] Bostwick, J. B. & Daniels, K. E. Capillary fracture of soft gels. *Phys. Rev. E* **88**, 042410 (2013). 10.1103/PhysRevE.88.042410 24229192

[22] Gang, P. & Severtson, S. J. Characterization of dynamic stick-and-break wetting behavior for various liquids on the surface of a highly viscoelastic polymer. *Langmuir* **24**(9), 4685–4692 (2008). 18442224 10.1021/la703790f

[23] Karpitschka, S., Das, S., van Gorcum, M., Perrin, H., Andreotti, B. & Snoeijer, J. H. Droplets move over viscoelastic substrates by surfing a ridge. *Nat. Commun.* (2015). 10.1038/ncomms8891 PMC4532859 26238436

[24] Newby, B. Z. & Chaudhury, M. K. Effect of interfacial slippage on viscoelastic adhesion. *Langmuir* **13**(6), 1805–1809 (1997).

[25] Wu-Bavouzet, F., Clain-Burckbuchler, J., Buguin, A., De Gennes, P.-G. & Brochard-Wyart, F. Stick-slip: wet versus dry. *J. Adhes.* **83**(8), 761–784 (2007).

[26] Style, R. W. et al. Liquid-liquid phase separation in an elastic network. *Phys. Rev. X* **8**(1), 011028 (2018).

[27] Zhao, M. et al. Growth and relaxation of a ridge on a soft poroelastic substrate. *Soft Matter* **14**(1), 61–72 (2018). 10.1039/c7sm01757j 29135008

[28] Jensen, K. E. et al. Wetting and phase separation in soft adhesion. *Proc. Natl. Acad. Sci. U.S.A.* **112**(47), 14490–14494 (2015). 26553989 10.1073/pnas.1514378112 PMC4664338

[29] Cai, Z., Skabeev, A., Morozova, S. & Pham, J.T. Fluid separation and network deformation in wetting of soft and swollen surfaces. *Commun. Mater.* **2**(21) (2021).

[30] Hauer, L., Cai, Z., Skabeev, A., Vollmer, D. & Pham, J. T. Phase separation in wetting ridges of sliding drops on soft and swollen surfaces. *Phys. Rev. Lett.* **130**(5), 058205 (2023). 36800444 10.1103/PhysRevLett.130.058205

[31] David Smith, J. et al. Droplet mobility on lubricant-impregnated surfaces. *Soft Matter* **9**(6), 1772–1780 (2013).

[32] Badr, R. G. M., Hauer, L., Vollmer, D. & Schmid, F. Cloaking transition of droplets on lubricated brushes. *J. Phys. Chem. B* **126**(36), 7047–7058 (2022). 36062355 10.1021/acs.jpcb.2c04640

[33] Zheng, S.-F. et al. Theoretical and three-dimensional molecular dynamics study of droplet wettability and mobility on lubricant-infused porous surfaces. *Langmuir* **39**(37), 13371–13385 (2023). 37675482 10.1021/acs.langmuir.3c02078

[34] Hourlier-Fargette, A., Antkowiak, A., Chateauminois, A. & Neukirch, S. Role of uncrosslinked chains in droplets dynamics on silicone elastomers. *Soft Matter* **13**(19), 3484–3491 (2017). 28440371 10.1039/c7sm00447h

[35] Wong, W. S. Y. et al. Adaptive wetting of polydimethylsiloxane. *Langmuir* **36**(26), 7236–7245 (2020). 32496071 10.1021/acs.langmuir.0c00538 PMC7346096

[36] Jerison, E. R., Ye, X., Wilen, L. A. & Dufresne, E. R. Deformation of an elastic substrate by a three-phase contact line. *Phys. Rev. Lett.* **106**(18), 186103 (2011). 21635105 10.1103/PhysRevLett.106.186103

[37] Seemann, R., Herminghaus, S. & Jacobs, K. Dewetting patterns and molecular forces: a reconciliation. *Phys. Rev. Lett.* **86**, 5534 (2001). 11415294 10.1103/PhysRevLett.86.5534

[38] Seemann, R., Herminghaus, S. & Jacobs, K. Shape of a liquid front upon dewetting. *Phys. Rev. Lett.* **87**, 196101 (2001). 11690430 10.1103/PhysRevLett.87.196101

[39] Becker, J. et al. Complex dewetting scenarios captured by thin film models. *Nat. Mater.* **2**, 59 (2003). 12652675 10.1038/nmat788

[40] Reim, D., Herminghaus, S. & Fery, A. Imaging of droplets of aqueous solutions by tapping-mode scanning force microscopy. *Ultramicroscopy* **69**(3), 211–2178 (1997).

[41] Herminghaus, S. & Pompe, T. Three-phase contact line energetics from nanoscale liquid surface topographies. *Phys. Rev. Lett.* **85**(9), 1930 (2000). 10970650 10.1103/PhysRevLett.85.1930

[42] Bommer, S. et al. Droplets on liquids and their journey into equilibrium. *Eur. Phys. J. E* **36**, 1–10 (2013). 23933987 10.1140/epje/i2013-13087-x

[43] Wang, Q. et al. Wetting-induced elastocapillary deformation of supported thin rubbery polymer films. *Macromolecules* **57**(21), 10112–10119 (2024).

[44] van Brummelen, E. H., Shokrpour Roudbari, M., Simsek, G. & van der Zee, K. G. Binary-fluid–solid interaction based on the Navier–Stokes–Cahn–Hilliard equations. *Fluid Struct. Interact.* **20**, 283–328 (2017).

[45] Aland, S. & Mokbel, D. A unified numerical model for wetting of soft substrates. *Int. J. Numer. Meth. Eng.* **122**(4), 903–918 (2021).

[46] Bhopalam, S. R., Bueno, J. & Gomez, H. Elasto-capillary fluid-structure interaction with compound droplets. *Comput. Methods Appl. Mech. Eng.* **400**, 115507 (2022).

[47] Schmeller, L. & Peschka, D. Sharp-interface limits of Cahn-Hilliard models and mechanics with moving contact lines. *Multiscale Model. Simul.* **22**(2), 869–890 (2024).

[48] Schmeller, L. & Peschka, D. Gradient flows for coupling order parameters and mechanics. *SIAM J. Appl. Math.* **83**(1), 225–253 (2023).

[49] Abels, H. (Non-)convergence of solutions of the convective Allen-Cahn equation. *Partial Differ. Equ. Appl.* **3**(1), 1 (2022).

[50] Andreotti, B. & Snoeijer, J. H. Soft wetting and the Shuttleworth effect, at the crossroads between thermodynamics and mechanics. *Europhys. Lett.* **113**(6), 66001 (2016).

[51] Logg, A., Mardal, K.-A. & Wells, G. *Automated Solution of Differential Equations by the Finite Element Method: The FEniCS Book* Vol. 84 (Springer Science & Business Media, 2012).

[52] Bhatia, Q. S., Chen, J.-K., Koberstein, J. T., Sohn, J. E. & Emerson, J. A. The measurement of polymer surface tension by drop image processing: application to PDMS and comparison with theory. *J. Colloid Interface Sci.* **106**(2), 353–359 (1985).

[53] Roe, R.-J. Surface tension of polymer liquids. *J. Phys. Chem.* **72**(6), 2013–2017 (1968).

[54] Souheng, W. Surface and interfacial tensions of polymer melts. II. Poly (methyl methacrylate), poly (n-butyl methacrylate), and polystyrene. *J. Phys. Chem.* **74**(3), 632–638 (1970).

[55] Dee, G. T. & Sauer, B. B. The molecular weight and temperature dependence of polymer surface tension: Comparison of experiment with interface gradient theory. *J. Colloid Interface Sci.* **152**(1), 85–103 (1992).

[56] Nose, T. Temperature dependence of interfacial tension of demixed polymer blend melts: polystyrene/poly(dimethylsiloxane)s. *Polym. J.* **29**(3), 218–223 (1997).

[57] Kreder, M. J. et al. Film dynamics and lubricant depletion by droplets moving on lubricated surfaces. *Phys. Rev. X*, **8**(031053) (2018).

[58] Lorenz, B. et al. Adhesion: role of bulk viscoelasticity and surface roughness. *J. Phys. Condens. Matter*. **25**(22) (2013). 10.1088/0953-8984/25/22/225004 23649298

[59] Masurel, R., Roché, M., Limat, L., Ionescu, I. & Dervaux, J. Elastocapillary ridge as a noninteger disclination. *Phys. Rev. Lett.* **122**(24), 248004 (2019). 31322373 10.1103/PhysRevLett.122.248004

[60] Qin, X., Style, R. W. & Dufresne, E. R. Surface elastic constants of a soft solid. *Soft Matter* **14**(6), 916–920 (2018). 29383365 10.1039/c7sm02431b

[61] Henkel, C. et al. Soft wetting with (a)symmetric Shuttleworth effect. *Proc. R. Soc. A*. **478**(2264) (2022). 10.1098/rspa.2022.0132 PMC9347665 35937429

[62] Liu, Z. S. Q. Osmocapillary phase separation. *Extreme Mech. Lett.* **7**, 27–33 (2016).

[63] Glover, J. D., McLaughlin, C. E., McFarland, M. K. & Pham, J. T. Extracting uncrosslinked material from low modulus Sylgard 184 and the effect on mechanical properties. *J. Polym. Sci.* **58**, 233–382 (2019).

[64] James, P. M., Barrall, E. M., Dawson, B. & Logan, J. A. Cross-linking of methyl silicone rubbers. Part ii. Analysis of extractables from samples cross-linked under various conditions. *J. Macromol. Sci.-Chem.* **8**(1), 135–155 (1974).

