# Supplementary material for: Exploring the gap between theory and experiment at the three-phase contact line of polystyrene droplets on soft PDMS
Source: Sci Rep. 2025 Dec 9;15:43486. doi: 10.1038/s41598-025-30195-y (PMC12695972; doi:10.1038/s41598-025-30195-y)
Supplement: Supplementary file 1 — Supplementary Information. [file 41598_2025_30195_MOESM1_ESM.pdf]

Supplemental Information

SI1 Characterization of Rheological Properties

The characteristic parameter describing the viscoelasticity of a material is the (complex) shear modulus $G = G' + iG$", where G' and G" are the storage and loss modulus, respectively. The (visco-) elasticity of the different PDMS substrates was characterized by a Haake-Mars-40 rheometer in plate-plate geometry (25 mm radius). The liquid PDMS mixtures were confined in the shear geometry and cured in place, at 80°C for 90 minutes, following the same protocol as for the preparation of the PDMS substrates that were used for the dewetting experiments. After the preparation of a sample, a frequency sweep test was carried out at room temperature in the range ($\omega = 0.1 - 100\,\mathrm{rad/s}$). For both tested PDMS mixtures, i.e. SG184 and SG186, we found a proportionality between the stress that is applied to the material and its strain response, and therefore a loss modulus G" that is negligible compared the storage modulus G'. Consequently one can consider $G = G'$ for SG184 and SG186, which are used here; the obtained results are summarized in the Table S1.

PDMS substrate	G (kPa)
Sylgard 184	595 ± 30
Sylgard 186	224 ± 30

Table S1: Shear modulus G of PDMS rubbers obtained by a Haake-Mars-40 shear rheometer in plate-plate geometry (25mm radius, $\omega = 0.1 - 100\,\mathrm{rad/s}$) at room temperature. The loss modulus was observed to be negligible, so $G = G'$ for SG184 and SG186.

SI2 Droplet Shape Characterization

The topography of the droplets was obtained by atomic force microscopy (AFM) in soft tapping mode, for both top and bottom sides using the lift-off technique explained in the methods section and here in Figure S1.

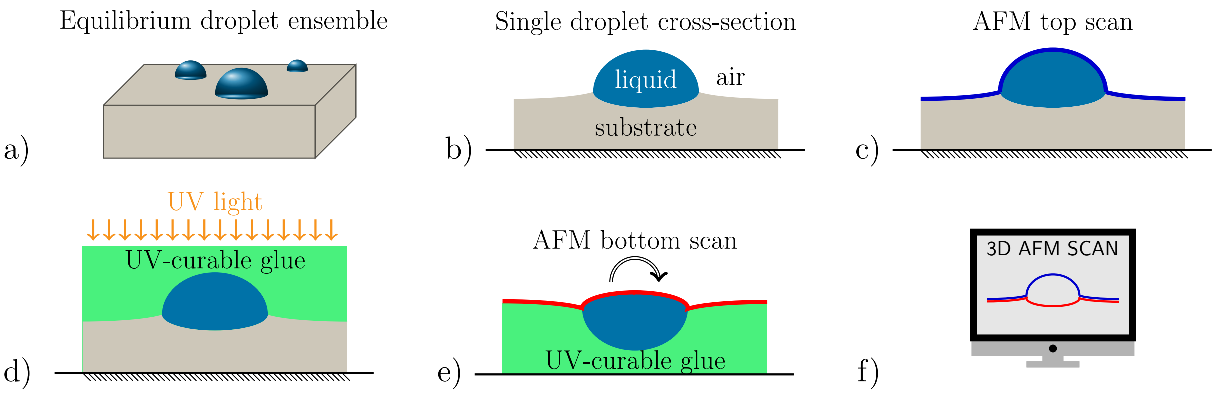

Figure S1: Sketch of experimental lift off process: a) Preparation of equilibrium droplet ensemble, b) consideration of cross-sections of single droplets, c) AFM top scan of PS/air and PDMS/air interface, d) covering the sample by UV-curable glue, e) peeling off the glue/PS from the PDMS substrate, flipping of the sample, and measurement of AFM bottom scan of formerly PS/PDMS and PDMS/air interface, f) composition of top and bottom AFM scan to a complete 3D droplet with postprocessing software.

The three-phase contact line (TPCL) was identified by contour analysis of the AFM profile, see Figure S2. The basis of this contour analysis is that the free PS-air surface has a constant curvature, and can be fitted by a circular arc, as shown in Figure S3. However, since the second derivative of point data, such as an AFM height contour, is extremely noisy, we instead use the first derivative from the AFM scans, where a sudden change in monotonicity indicates the exact position of the TPCL in the cross-section, as shown in Figure S2. One step of smoothing, involving the nearest two scanlines, was applied to slightly reduce the noise in the first derivative, allowing the resulting position of the TPCL to be determined with an accuracy of ±1 px relative to the AFM scan resolution.

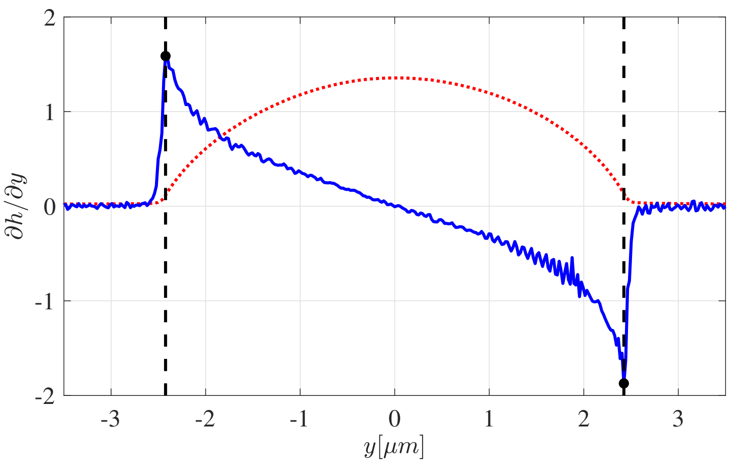

Figure S2: Droplet cross-section on SG184 obtained with AFM (red dots) with corresponding 1st derivative (blue solid). The three-phase contact line can be clearly identified by the sharp local minimum, respectively maximum in the 1st derivative.

The determination of the TPCL using this strategy was proven to be very robust and was also used on cross sections of all the presented samples. Alternatively, it would be also possible in principle to determine the TPCL via the phase contrast of the AFM, which indicates a material contrast. However, the phase contrast on those samples imaged in soft tapping is not very robust and precise, e.g. due to slight contamination of the AFM tip, and is thus not used here.

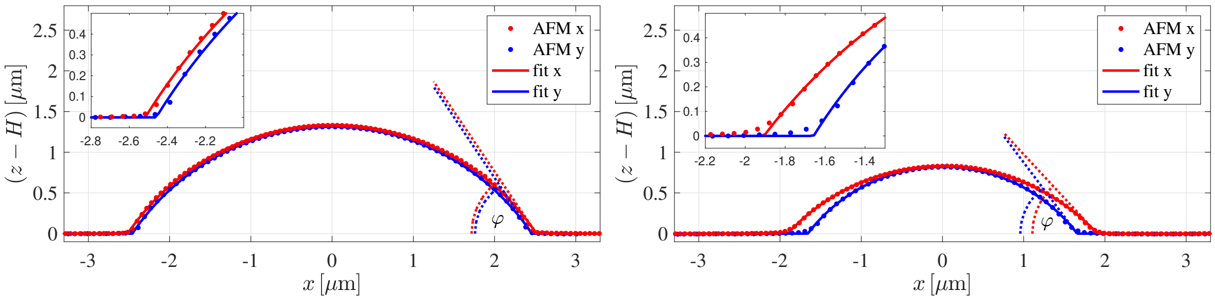

Figure S3: Circular arc fits to AFM cross sections in two perpendicular directions for droplets on (left) SG184 and (right) SG186 PDMS substrates, where Young contact angles θ are indicated.

AFM images of polystyrene (PS) droplets on SG184 and 186 substrates are shown in Figure S3 together with circular arc fits along the smallest and largest axis of the drop. The droplet radius on SG184 varies by 3%, i.e. within the accuracy of the AFM, whereas the drop radii on SG186 vary by 13% between both measurement directions indicating an elliptical drop shape that is not fully equilibrated but also does not equilibrate further on experimentally achievable time scales, as described in more detail in the main text. Accordingly, the contact angle on the well equilibrated PS droplets of SG184, $\theta = (55.9 \pm 0.3)^\circ$ has only a small uncertainty. For PS droplets on SG186, instead, we observe stronger deviations from an axisymmetrical droplet shape, so that the contact angle for the largest droplets vary between $\theta = 47.0^\circ$ (long axis) and $\theta = 52.7^\circ$ (short axis).

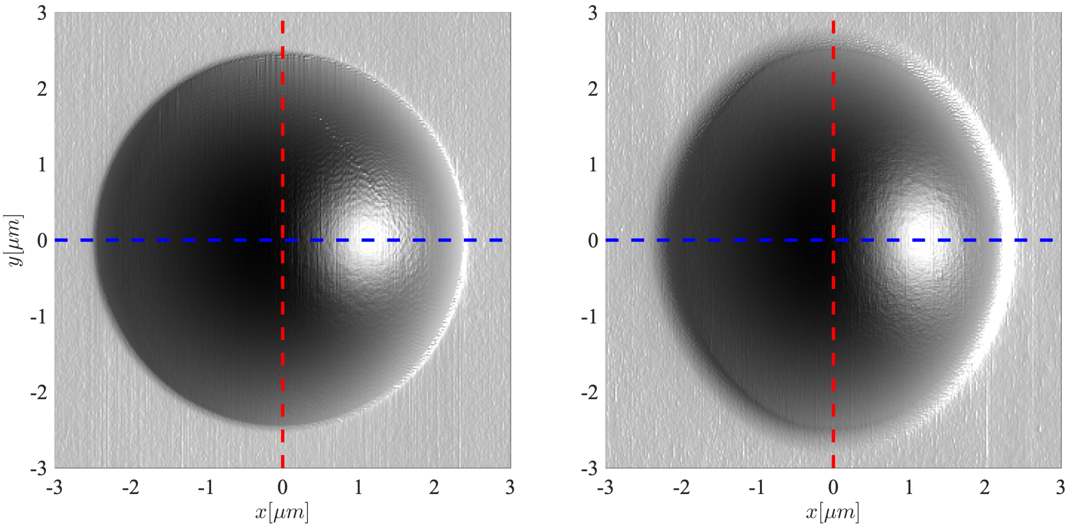

Figure S4: AFM topside scans of PS droplets on (left) SG184 and (right) SG186 substrates that were shown in Figure S3. The PS droplet on SG184 exhibits rotational symmetry, whereas the droplet on SG186 shows a noticeable ellipticity. Gray shading based on height and lighting based on slope to improve visibility of droplet shape. The dashed lines indicate the cross-sections used in Figure S3.

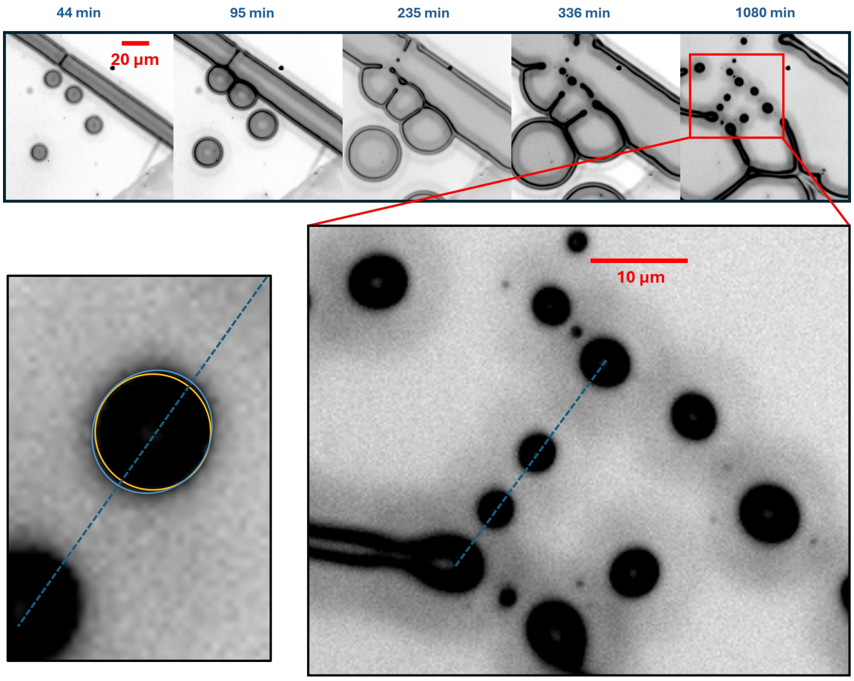

Figure S5: Optical microscopy time series of a PS 17.8 kg/mol film (thickness $h = 120nm$) dewetting from SG186 [1:10] substrate. The annealing time at $T = 120°\text{C}$ is given in each frame. The bottom right image is a close up after 18 hours of dewetting, the dashed blue line indicates the long axis of the droplets that is aligned with the previous dewetting ribbon. Bottom left shows a close up of the droplet situated in the middle of the dashed line, where the contour of the droplet is delimited by a blue ellipse, while a circular fit based on the short axis is indicated in yellow, the elliptical shape of the droplets exhibits a long axis about 10% longer than the short axis.

Based on these differences, we can estimate the contact angle hysteresis for PS on SG184 and SG186. To understand the physical justification for that let's revisit the pathway of the dewetting process leading to droplets, which is displayed in Figure S5: First holes pop up in an initially uniform thin film. As these holes grow, the dewetted liquid is collected in a rim surrounding the hole. When neighboring holes become large enough and their dewetting rims touch each other, the rims merge and form a (pretty) straight liquid ribbon that has a circular cross section. This straight liquid ribbon will decay via (a generalized) Rayleigh-Plateau instability into single droplets. When this straight ribbon decays into smaller segments, droplets are formed when each segment of the liquid ribbon retracts in length and grows in width until eventually circular holes are formed. So based on this pathway, some parts of the droplet are formed from a (rather) receding three phase contact line (TPCL) while others are formed from a (rather) advancing TPCL. The observation (within experimental accuracy) of 'perfectly' round PS droplets on SG184 substrates with constant contact angle of $\theta = (55.9 \pm 0.3)°$, thus suggests a situation very close to equilibrium with no noticeable difference between receding and advancing contact angle. The situation in case of PS dewetting on SG186, however, is slightly different as the droplets are slightly elliptically deformed. The long axis of these ellipses are aligned in direction of the previously straight dewetting ribbons and correspond to a receding contact angle of about $\theta = 47.0°$. The contact angle along the short axis correspond to an advancing contact angle of $\theta = 52.7°$. So based on these differences we could estimate a contact angle hysteresis for PS on SG186 of $\Delta\theta \approx \pm 3°$. Furthermore, the observed

globally random orientation of the elliptical drop base, which locally reflects the history of the de-wetting process, rules out global pre-stressing of the PDMS as a possible reason for this alignment.

SI3 Surface Chemistry Characterization

Section SI2 shows how the topography signals obtained by AFM allow to precisely identify the contours of PS droplets. However, this technique is based on the physical response of the material and does not give precise information about the chemical composition of the probed surface. To explore the chemical composition at the surface of the PS droplets, **NanoIR** (Bruker) was applied, Figure S6 (left). The NanoIR technique is based on a pulsed and tunable IR laser focused on the sample and synchronized with the AFM tapping frequency. When the applied wavelength matches an absorbance band of the substrate, it causes a local thermal expansion of the surface that is detected by the AFM tip. By this strategy, spatially resolved infrared absorption spectra are obtained that can be correlated with the presence of specific molecular bands, giving precise information about the molecular composition of the probed surface. However, the surface sensitivity of NanoIR is not precisely known, but we can assume that the dominant signal comes from a depth below 100 nm.

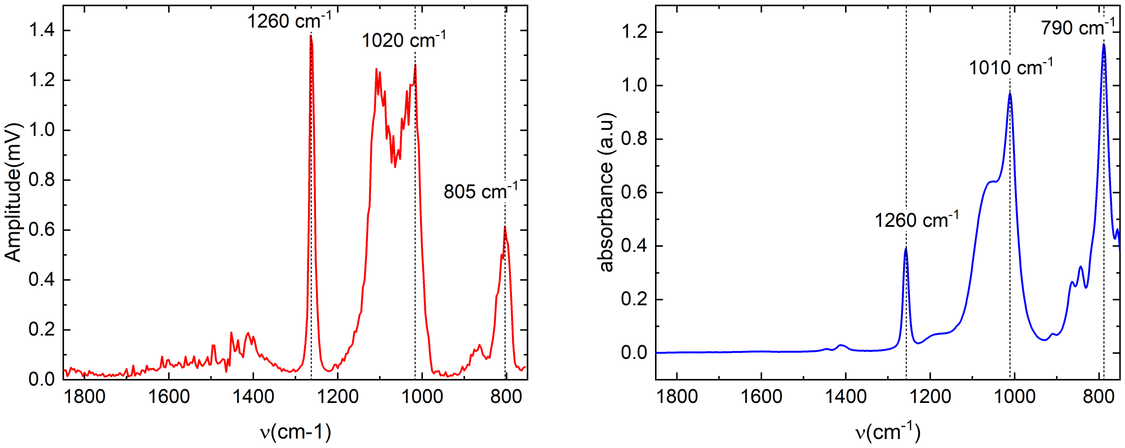

Figure S6: (left) NanoIR (Bruker) absorption spectrum measured on top of a PS droplet showing the presence of PDMS peaks. (right) IR absorption spectrum Obtained by ATR-FTIR for crosslinked SG184.

In Figure S6 (right), the infrared spectra of a crosslinked bulk SG184 sample obtained by attenuated infrared spectroscopy (ATR-FTIR) is shown for comparison. Both spectra show three peaks that are characteristic for PDMS (a peak at 1260 cm^{-1} and at 805 cm^{-1} for the Si–$(CH_3)_3$ band and Si–CH_3 and a peak at 1020 cm^{-1} for Si–O–Si) and thus reveal the presence of a thin layer of PDMS on top of the PS droplet. This observation is in line with expectations from the positive spreading coefficient favoring the cloaking of the PS-droplet by PDMS in order to lower its surface tension.

SI4 Axisymmetric Droplet Relaxation Model

Below, we detail the space and time discretization for the sharp-interface model that describes droplet relaxation via the deformation $\chi(t)$ as $t \to \infty$. This algorithm's implementation achieves the necessary robustness and precision to predict stationary axisymmetric droplets for a range of droplet radii relevant to the capillary length. The initial shape of the domain Ω^0 is illustrated in the manuscript in Figure 3.

Assuming axisymmetry, we replace three-dimensional volume and surface measures by $\mathrm{d}\boldsymbol{x} = 2\pi r\,\mathrm{d}r\,\mathrm{d}z$ and $\mathrm{d}\boldsymbol{a} = 2\pi r\,\mathrm{d}s$ and define the axisymmetric deformation gradient

$$\boldsymbol{F} = \nabla\boldsymbol{\chi} := \begin{pmatrix} \partial_r\chi_r & 0 & \partial_z\chi_r \\ 0 & r^{-1}\chi_r & 0 \\ \partial_r\chi_z & 0 & \partial_z\chi_z \end{pmatrix}$$

and rewrite the energy (2) and the evolution in (4) correspondingly. Following [64, 47], we discretize (4) by using P_2 finite elements for the deformation χ and use an incremental minimization strategy to discretize in time and ensure discrete energy descent. The boundaries of the subdomains Ω_i are assumed polygonal and resolved by the edges of the computational triangular mesh $\Omega_h = \cup_{n=1}^{N_{\text{element}}} T_h$. Near the contact line we employ a heuristic spatial refinement procedure in order to resolve singular elastic deformations due to the capillary ridge at the TPCL caused by the interfacial forces. The implementation of the nonlinear problem is provided in FEniCS [50].

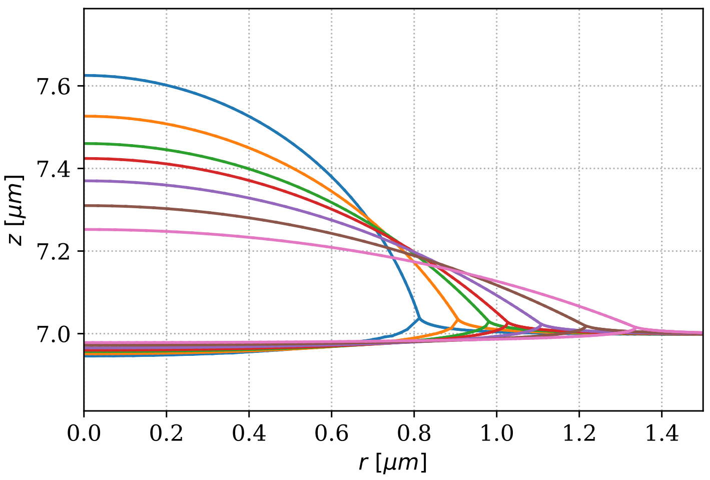

Figure S7: Stationary droplet shapes for different initial radii $R = r_x$ corresponding to Figure S8. Note that the red full lines corresponds to the energy minimizer in Figure S8 and corresponds approximately to the parameters of the middle droplet on SG186 in Figure 5.

In the dynamic relaxation model, we impose no-slip boundary conditions, i.e. the displacements and velocities along the substrate-liquid interface are continuous. This leads to a pinning of the contact line in the sense that material points on each side close to the PS-PDMS interface move jointly and the displacement of the contact line also generates a (singular) elastic energy. Therefore, the equilibrium state depends on the chosen initial data and does not minimize the free energy in the space of all admissible shapes, e.g., see the different stationary droplet shapes in Figure S7.

To achieve the global minimal energy for an axisymmetric droplet, we vary the initial shape of the liquid domain Ω_ℓ^0 and therefore the position of the initial contact line by

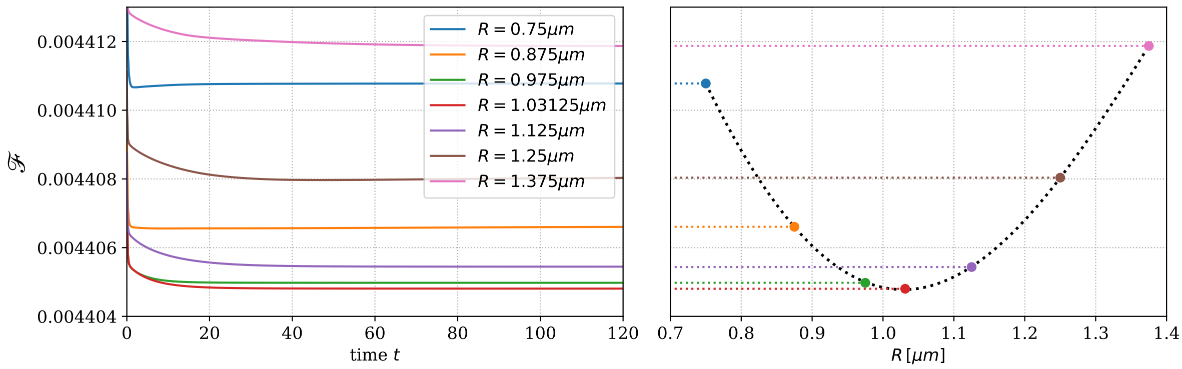

Figure S8: (left) Energy evolution $\mathscr{F}(t)$ of droplet configurations approaching stationary states for different initial radii $R = r_x$ and (right) corresponding stationary energies as a function of droplet radius R. The black dotted line is a fitted 4th order polynomial.

choosing the initial radius r_x so that $r_z = r_z(V)$ is determined by the given droplet volume V. To match an individual AFM measurement, the droplet volume V is determined from the experimental data and we obtain the values stated in Table S2. We vary the initial radius r_x, compute the resulting stationary shape as $t \to \infty$ in the left panel of Figure S8, and compute its equilibrium free energy as a function of r_x as shown in the right panel of Figure S8. Usually we compute the energy of stationary states only for a few r_x values, e.g., 7 values in Figure S8, and find the minimum by interpolating with a polynomial. This optimal radius r_x is used to compute the optimized droplet shape – this is the shape to which the dynamics would have converged with the more admissible boundary condition at the PS-PDMS interface.

PDMS	volume [μm^3]	radius [μm]
SG184	20.8 ± 0.5	2.804
SG184	2.16 ± 0.02	1.314
SG184	0.147 ± 0.008	0.512
SG186	15.1 ± 0.1 (10.61 ± 0.1)	2.676
SG186	0.85 ± 0.01 (0.60 ± 0.04)	0.975
SG186	0.042 ± 0.002 (0.027 ± 0.005)	0.338

Table S2: Computed volumes and radius of (assumed axisymmetric) PS droplets shown in Figure 5. Values in brackets for SG186 are based on short axis.

SI5 Dependence on Solid Angle

In this work, we employ the hybrid construction (6) to obtain surface tensions compatible with the observation of cloaking and the Young angle for large droplet sizes. However, it has been noted in the literature that, particularly for small or vanishing solid angles, defining and measuring the Neumann angles $\vartheta_\ell, \vartheta_s$ can be somewhat problematic due to the singular nature of the elastocapillary ridge [20]. We verify and confirm this observation by showing in Figure S9 droplets with radii $R \approx 2\,\mu\mathrm{m}$ and surface tensions corresponding to the same Young angle but two solid angles, $\vartheta_\mathrm{s} = 0°$ and $\vartheta_\mathrm{s} = 40°$. On the droplet scale, the interface shapes appear identical, and only at scales of $\pm 5\,\mathrm{nm}$ are small deviations in angle and height observable, well below the resolution of realistic AFM measurements.

This leads us to conclude that, in the moderately soft limit for $R \ll \lambda_\mathrm{c}$ and small opening angle ϑ_s, the Neumann angle can only be observed at scales smaller than the elastocapillary length λ_c and potentially even at or below molecular scales $a \sim 10^{-9}\,\mathrm{m}$.

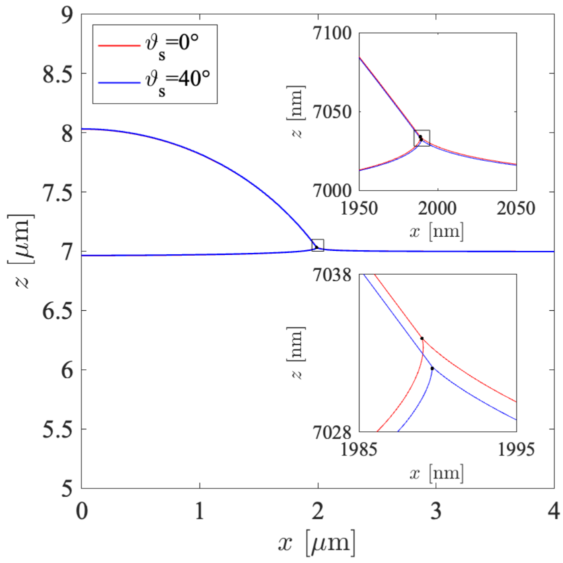

Figure S9: Comparison of numerical solutions for solutions of sharp-interface model with surface tensions with the same Young angle but different solid opening angles ϑ_s.

SI6 Dependence on Substrate Thickness

The experiments and simulations presented in this paper were performed for moderately soft droplets $R \ll \lambda_\mathrm{c}$ on moderately thick substrates, where the radius of the largest droplets is comparable to the substrate thickness $H = 7\,\mu\mathrm{m}$. To estimate the effect of substrate thickness, we conducted a small study on such a droplet, see Figure S10. The inset of the figure shows that, within the range $H = 5$–$10\,\mu\mathrm{m}$, the indentation depth at the PS-PDMS interface, approximately 50 nm, changes only by about $\pm 5\,\mathrm{nm}$. This variation is comparable to the experimental interface roughness. Furthermore, the position and elevation of the TPCL and the wetting ridge are not visibly affected by the substrate thickness. Therefore, variations in substrate thickness do not account for the observed discrepancies near the TPCL.

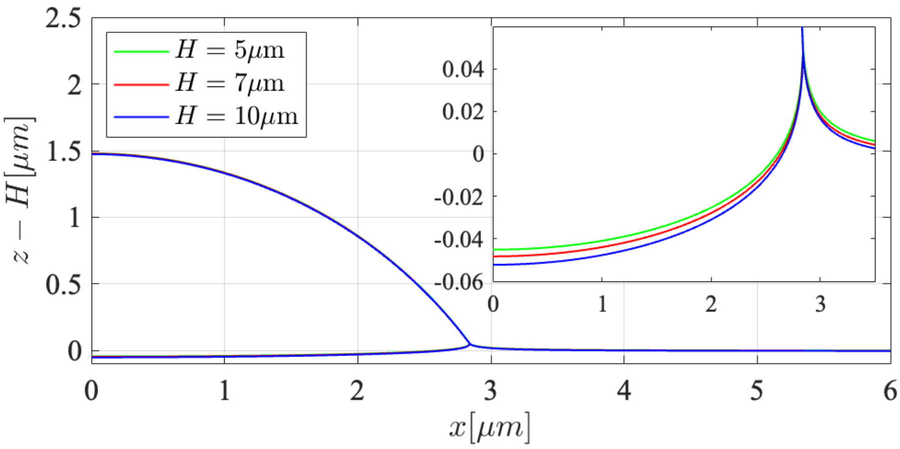

Figure S10: Numerical comparison of three substrate heights, where the base height is subtracted from the z coordinate.

SI7 Magnitude of displacement near TPCL

In order to estimate the potential impact of the Shuttleworth effect, we display $\sqrt{\mathrm{tr}(\boldsymbol{F}^T\boldsymbol{F})/3}$ as a simple local measure of stretching to estimate the size of spatial regions where surface tension and energy could potentially deviate from each other. The shown numerical solution in Figure S11 corresponds to a droplet with radius $R \sim 1\,\mu\mathrm{m}$, where the back bar indicates 100 nm, which is comparable to the elastocapillary length $\lambda_c = 80\,\mathrm{nm}$ for SG186. The region where the local stretching exceeds 10%, highlighted by lighter colors near the TPCL, is clearly much smaller than λ_c and restricted to a scale of about 10 nm.

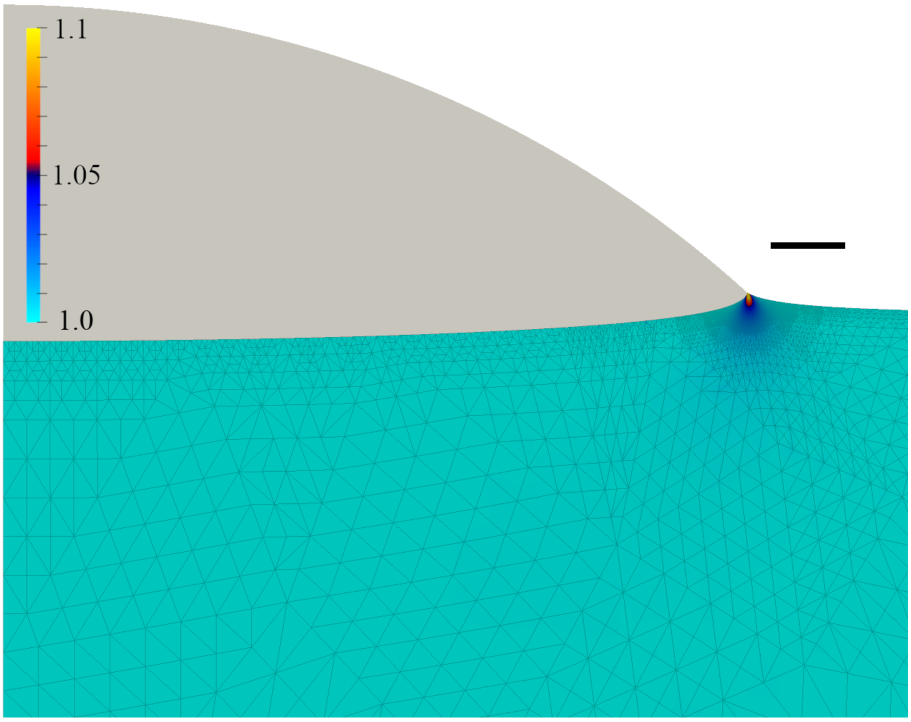

Figure S11: For a micrometer sized droplet we show $\sqrt{\mathrm{tr}(\boldsymbol{F}^T\boldsymbol{F})/3}$ to indicate the relative local stretching near the TPCL. The black bar indicates 100nm length scale.

SI8 Dependence on PDMS Shear Modulus

In the manuscript, it is argued that the observed discrepancies, particularly the enhanced elevation of the TPCL, can only be explained by a locally enhanced elastocapillary length. Since we also argue that a global variation in the shear modulus is necessary to achieve good agreement with global droplet shapes, one might consider the possibility of matching shapes using a globally increased elastocapillary length λ_c. In Figure S12, we compare theoretical and experimental profiles for a drastically reduced PDMS shear modulus in SG186 $\frac{1}{16}G_{\mathrm{SG186}}^{\mathrm{exp}}$. While this reduction predicts a similar elevation of the TPCL as observed in the experiment, the theoretical global droplet profile deviates significantly from the experimentally measured profile, thereby excluding the possibility of global variations in the shear modulus as an explanation.

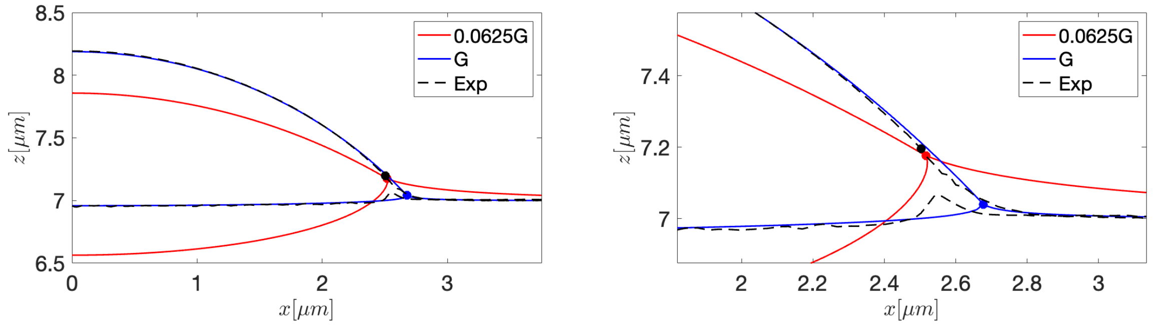

Figure S12: Comparison of experimental AFM cross section for SG186 (black dashed line) and position of contact line (black dot) compared to theoretical predictions, global shape (left), about three times magnified (right). The numerical shape and TPCL are shown in blue for the correct shear modulus $G = G_{\mathrm{SG186}}$, while the data in red color display the shape computed for much softer PDMS substrate with shear modulus $\frac{1}{16}G_{\mathrm{SG186}}^{\mathrm{exp}}$.
